# First-Principles Insights into the Relative Stability, Physical Properties, and Chemical Properties of MoSe_2_

**DOI:** 10.1021/acsomega.2c08217

**Published:** 2023-04-05

**Authors:** Lathifa Banu S, Vasu Veerapandy, Helmer Fjellvåg, Ponniah Vajeeston

**Affiliations:** † Department of Computational Physics, School of Physics, Madurai Kamaraj University, Palkalai Nagar, Madurai 625021, Tamil Nadu, India; ‡ Department of Chemistry and Center for Materials Science and Nanotechnology, University of Oslo, Oslo 0371, Norway

## Abstract

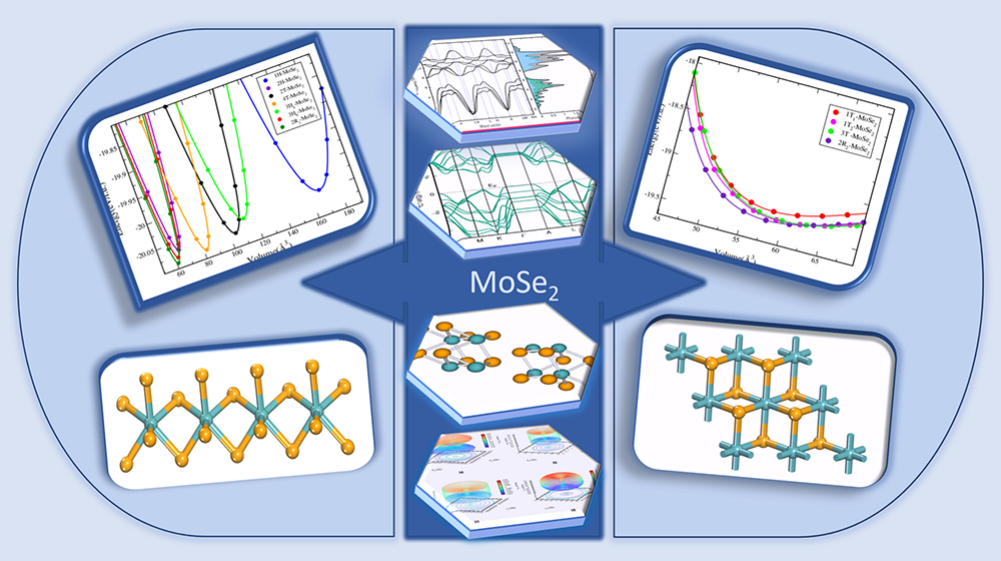

A fascinating transition-metal dichalcogenide (TMDC) compound, MoSe_2_, has attracted a lot of interest in electrochemical, photocatalytic, and optoelectronic systems. However, detailed studies on the structural stability of the various MoSe_2_ polymorphs are still lacking. For the first time, the relative stability of 11 different MoSe_2_ polymorphs (1H, 2H, 3H_a_, 3H_b_, 2T, 4T, 2R_1_, 1T_1_, 1T_2_, 3T, and 2R_2_) is proposed, and a detailed analysis of these polymorphs is carried out by employing the first-principles calculations based on density functional theory (DFT). We computed the physical properties of the polymorphs such as band structure, phonon, and elastic constants to examine the viability for real-world applications. The electronic properties of the involved polymorphs were calculated by employing the hybrid functional of Heyd, Scuseria, and Ernzerhof (HSE06). The energy band gap of the polymorphs (1H, 2H, 3H_a_, 3H_b_, 2T, 4T, and 2R_1_) is in the range of 1.6–1.8 eV, coinciding with the experimental value for the polymorph 2H. The covalent bonding nature of MoSe_2_ is analyzed from the charge density, charge transfer, and electron localization function. Among the 11 polymorphs, 1H, 2H, 2T, and 3H_b_ polymorphs are predicted as stable polymorphs based on the calculation of the mechanical and dynamical properties. Even though the 4T and 3H_a_ polymorphs’ phonons are stable, they are mechanically unstable; hence, they are considered to be under a metastable condition. Additionally, we computed the direction-dependent elastic moduli and isotropic factors for both mechanically and dynamically stable polymorphs. Stable polymorphs are analyzed spectroscopically using IR and Raman spectra. The thermal stability of the polymorphs is also studied.

## 1 Introduction

TMDCs are a novel luminaire building material that has recently gained much attention.^1,2^ A wide range of two-dimensional and few-layered TMDCs have been created for a variety of applications including energy storage, catalysis, optoelectronics, and microelectronics.^3^ Especially, more than 90% of tumor cells were destroyed using phototherapy medications based on TMDC materials including MoS_2_, WS_2_, and MoSe_2_. Multiple polymorphs exist in TMDCs due to metal coordination and monolayer stacking order already reported by Donarelli et al.^3,4^ According to crystal symmetry, TMDCs naturally hold the T (tetragonal), H (hexagonal), and rare R (rhombohedral) phases. Layers of TMDCs have been adjusted according to several works in many different applications.

Among the TMDC materials that have been described earlier, MoSe_2_ (molybdenum diselenite) is one of the finest materials. It has a greater electrical conductivity than MoS_2_ when compared.^5,6^ For the bilayer, MoSe_2_ exhibits a direct band gap, which switches to an indirect band gap as the layers rise.^7^ When subjected to NIR (near infrared) laser light, MoSe_2_ has been explored as a nonphotothermal agent. Photothermal experiments have just recently been documented by Yuwen et al.^6^ The resultant monolayer MoSe_2_ is used as a biosensor for detecting VOCs (volatile organic compounds) in lung cancer, especially when Al is doped with MoSe_2_ (AlMoSe_2_), which have already been reported by Liu et al.^8^ MoSe_2_ materials can be used in both photodetectors and switchable transistors. MoSe_2_ is a novel material that can absorb gas molecules on its surfaces, assisting in the charge transfer process. The MoSe_2_ nanosheet is one of the best gas sensors, detecting NO_2_ even at ambient temperature, as Chen et al. described before.^9,10^ As previously stated, single-layer MoSe_2_ based on ammonia is used as a gas sensor.^11^ In a recent article, the N-doped carbon at MoSe_2_ core/branch nanostructure displayed outstanding lithium storability reported by Morales et al.^12^ A covalent bond between atomic layers create the usual structure of MoSe_2_, with a single Mo atomic layer sandwiched between two Se atomic layers.^13^ The stacking sequence and/or layer spacing can be changed to modify the MoSe_2_ structure.^14^ Depending on the structure, different MoSe_2_ polymorphs can be created, although not all of them will be stable. A stability study is required before using polymorphs for a variety of applications.

In this study, to find the stability impact, we conducted in-depth theoretical assessments on features for 11 different MoSe_2_ polymorphs using DFT calculations for the first time.^15,16^ Stability investigation was carried out beforehand by finding the low energies of all polymorphs, as well as coordinate configurations were found for various layers of MoSe_2_ polymorphs.^17^ The electronic characteristics of MoSe_2_ polymorphs are examined. From the electronic properties, we observed that seven polymorphs are semiconductors in nature and four polymorphs are metallic in nature.^18^ To investigate how the polymorphs bind, the charge density, charge transfer, and electron localization function are also estimated. The phonon and mechanical properties of all 11 polymorphs are extensively investigated to predict the stable nature of each polymorph. Six polymorphs are dynamically stable, and the remaining five polymorphs are dynamically unstable. The novel IR and Raman spectra are estimated for stable structures, which are used for vibrational study and to characterize the structure of polymorphs. In addition, the thermal stability of the polymorphs was examined by the thermal characteristics of the polymorphs. The primary purpose of this research was to analyze the structural, mechanical, dynamic, and thermal stability of different MoSe_2_ polymorphs. From this development, novel stable polymorphs are found, which might be used in quite a lot of applications like energy storage, catalysis, optoelectronics, and microelectronics for future purposes.

## 2 Results and Discussion

### 2.1 Structure and the Stability of MoSe_2_ Polymorphs

**Figure 1:**
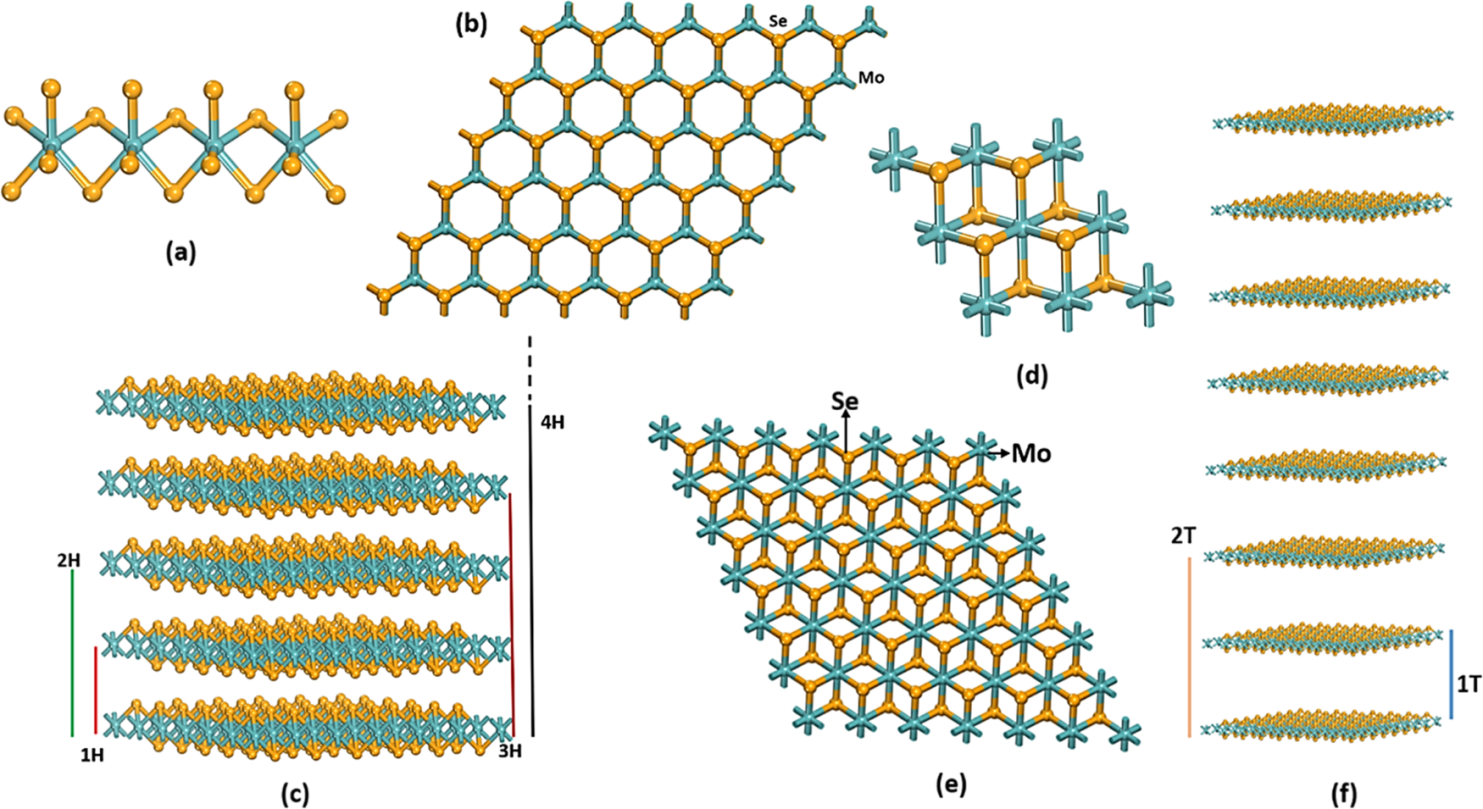
(a) Basic building blocks and (b) the planar view of the Mo–Se trigonal prismatic phase. (c) Stacking sequences of MoSe_2_ hexagonal polymorphs. (d) The basic building blocks and (e) the planar view of the MoSe_2_ octahedral prismatic phase. (f) Stacking sequences of MoSe_2_ trigonal polymorphs.

The Mo atom is sandwiched between two Se atoms (Se–Mo–Se) creating the MoSe_2_ structure. The intralayer of the MoSe_2_ structure will be two-dimensionally covalently bonded, whereas the interlayer will be weakly coupled due to the weak van der Waals-type forces.^19^ MoSe_2_ featured many polymorphs depending on the layer stacks.^20^ A single layer of MoSe_2_ will have a trigonal prismatic phase with a hexagonal structure and an octahedral prismatic phase with a trigonal structure (Figure 1). In the octahedral prismatic phase, the metal atoms are octahedrally coordinated by six nearby Se atoms. In the trigonally prismatic phase, the metal atoms are trigonally coordinated by two nearby Se atoms. Variations in the stacking order and registry of succeeding Se–Mo–Se sandwiches of the hexagonal and trigonal structure along the *c*-axis give rise to several crystal polymorphs or polytypes in three dimensions (Figure 1).^20^ In this work, based on the trigonal prismatic phase and octahedral prismatic phase, 11 different polymorphs are formed (1H-MoSe_2_, 2H-MoSe_2_, 1H_a_-MoSe_2_, 1H_b_-MoSe_2_, 2T-MoSe_2_, 4T-MoSe_2_, 2R_1_-MoSe_2_, 1T_1_-MoSe_2_, 1T_2_-MoSe_2_, 3T-MoSe_2_, and 2R_2_-MoSe_2_). In this architecture, the integer defines the number of layers per unit cell along the *c*-axis. Trigonal, hexagonal, and rhombohedral structural symmetries are denoted by the letters T, H, and R, respectively.^20−22^

In this work, polymorphs are separated into two groups (group A and group B) based on the energies of the polymorphs. In group A, we observe that two polymorphs are trigonal, one polymorph is rhombohedral and the other four are hexagonal (1H-MoSe_2_, 2H-MoSe_2_, 1H_a_-MoSe_2_, 1H_b_-MoSe_2_, 2T-MoSe_2_, 4T-MoSe_2_, and 2R_1_-MoSe_2_).^23^ In group B, one of the polymorphs is rhombohedral, while three are trigonal structures (1T_1_-MoSe_2_, 1T_2_-MoSe_2_, 3T-MoSe_2_, and 2R_2_-MoSe_2_). The bonding length of Mo–Se and the structure formation of all of the polymorphs are given in the Supporting Information on Page S2.

**Figure 2:**
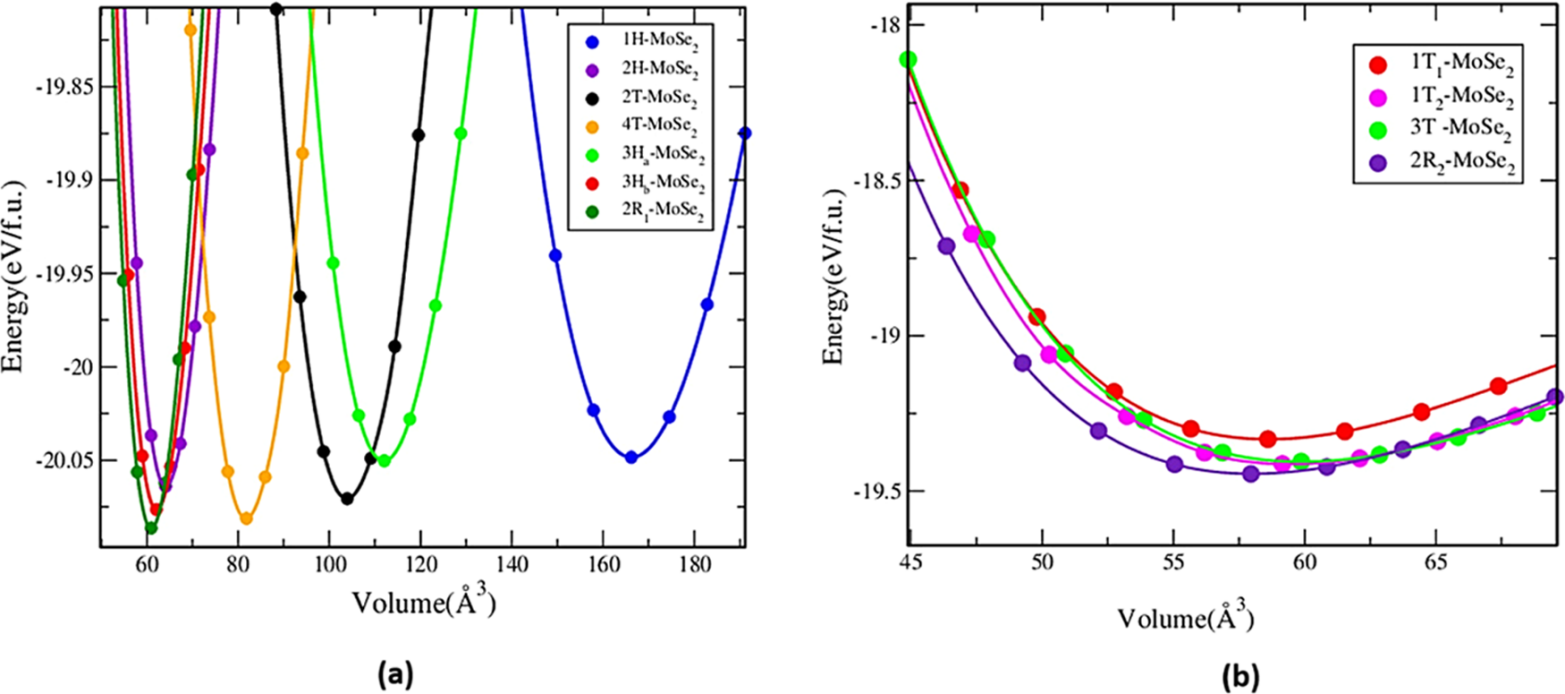
Total energy vs volume curve for (a) group A and (b) group B. Calculated total energy as a function of the volume of the unit cell for the different MoSe_2_ polymorphs.

The relative stability of the polymorphs is investigated after structural optimization. In our simulation, the total energy of all polymorphs as a function of volume with varying ranges is noticed.^18^ From Figure 2a, all of the polymorphs in group A show that the energies are very close for different volume ranges, which confirms that MoSe_2_ can easily be found in any of these variants. Figure 2b shows that group B polymorphs have low energy between −19.33 and −19.44 eV/f.u., which is lower than that of group A. The low energy of group A and group B are given in Table S1 of the Supporting Information. The formation energy of group A is in the range of −2.13 to −2.17 eV/f.u. and group B obtained −1.43 to −1.5 eV/f.u. range of formation energy. The volume, the minimum energy, and the formation energy of all of the polymorphs are given in Table S1 of the Supporting Information on Page S1.

The calculated positional and lattice constants of different polymorphs are given in Table 1. Experimental results (*a* = *b* = 3.288 Å, *c* = 12.900 Å) well coincide with the 2H-MoSe_2_ polymorph, which has already been reported by Brixner et al.^22^ The lattice constant of 2H-MoSe_2_ (*a* = *b* = 3.269 Å, *c* = 13.857 Å) well coincided with the previous result (*a* = *b* = 3.28 Å, *c* = 12.91 Å) given by Silvestrelli and Ambrosetti.^19,24^

**Table 1:** Unit Cell Constants and Coordinates for the Investigated MoSe_2_ Polymorphs, Space Group, and Material Project IDs Are Mentioned Near the Polymorphs, in Parentheses

polymorph	unit cell constants (Å)	atom	site	*X*	*Y*	*Z*
1H-MoSe_2_ (*P*6̅*m*2; mp-1023924)	*a* = *b* = 3.267, *c* = 17.975	Mo1	1a	0	0	0
		Se1	2i	2/3	1/3	0.0920
2H-MoSe_2_ (*P*6_3_/*mmc*; mp-1018809)	*a = b* = 3.269, *c* = 13.857	Mo1	2b	0	0	1/4
	*a = b* = 3.280, *c* = 12.910^22^	Se1	4f	2/3	1/3	0.3698
	*a = b* = 3.280, *c* = 12.920^24^					
	*a* = *b* = 3:299, *c* = 12.938^19^					
3H_a_-MoSe_2_ (*P*6̅*m*2; mp-1025874)	*a* = *b* = 3.267, *c* = 36.342	Mo1	2i	2/3	1/3	0.7684
		Mo2	1c	1/3	2/3	0
		Se1	2i	2/3	1/3	0.0458
		Se2	2h	1/3	2/3	0.2773
		Se3	2h	1/3	2/3	0.8139
3H_b_-MoSe_2_ (*P*6_3_/*mmc*; mp-2815)	*a* = *b* = 3.268, *c* = 13.425	Mo1	2d	2/3	1/3	1/4
		Se1	4f	2/3	1/3	0.8737
2T-MoSe_2_ (*P*3̅*m*1; mp-1023939)	*a* = *b* = 3.268, *c* = 22.471	Mo1	2b	0	0	1/4
		Se1	4f	2/3	1/3	0.3698
4T-MoSe_2_ (*P*3̅*m*1; mp-1027525)	*a* = *b* = 3.269, *c* = 35.382	Mo1	2d	2/3	1/3	0.0935
		Mo2	2d	2/3	1/3	0.7211
		Se1	2d	2/3	1/3	0.3258
		Se2	2d	2/3	1/3	0.9535
		Se3	2d	2/3	1/3	0.2317
		Se4	2d	2/3	1/3	0.8590
2R_1_-MoSe_2_ (*R*3̅*m*; ICSD_31067; 1434)	*a* = *b* = 3.272, *c* = 19.713	Mo1	1a	0.9997	0.9997	0.9997
		Se1	1a	0.4175	0.4175	0.4175
		Se2	1a	0.2490	0.2490	0.2490
1T_1_-MoSe_2_ (*P*3̅*m*1; mp-147)	*a* = *b* = 3.326, *c* = 15.451	Mo1	1a	0	0	0
		Se1	2d	2/3	1/3	0.2667
1T_2_-MoSe_2_ (*P*3̅; mp*-*164)	*a* = *b* = 3.238, *c* = 6.510	Mo1	1a	0	0	0
		Se1	2d	2/3	1/3	0.2652
3T-MoSe_2_ (*R*3̅*m*; mp-1558544)	*a* = *b* = 3.226, *c* = 19.282	Mo1	1b	1/2	1/2	1/2
		Se1	2c	0.2561	0.2561	0.2561
2R_2_-MoSe_2_ (**P**3**m**1; mp-11238797)	*a* = *b* = 3.240, *c* = 6.584	Mo1	1b	0	0	1/2
		Se1	2d	2/3	1/3	0.2383

### 2.2 Electronic Structure

A detailed investigation of the electronic computations is performed for all computed MoSe_2_ polymorphs. We used HSE06 for the electronic calculation in our study instead of GGA, which gave us improved accuracy. Materials with semiconducting qualities can absorb visible light, although metals can be employed as conductors. In this case, HSE06 band gap calculations allow us to understand the polymorphs in group A as semiconductors and the group B polymorphs as metals.^25^

**Figure 3:**
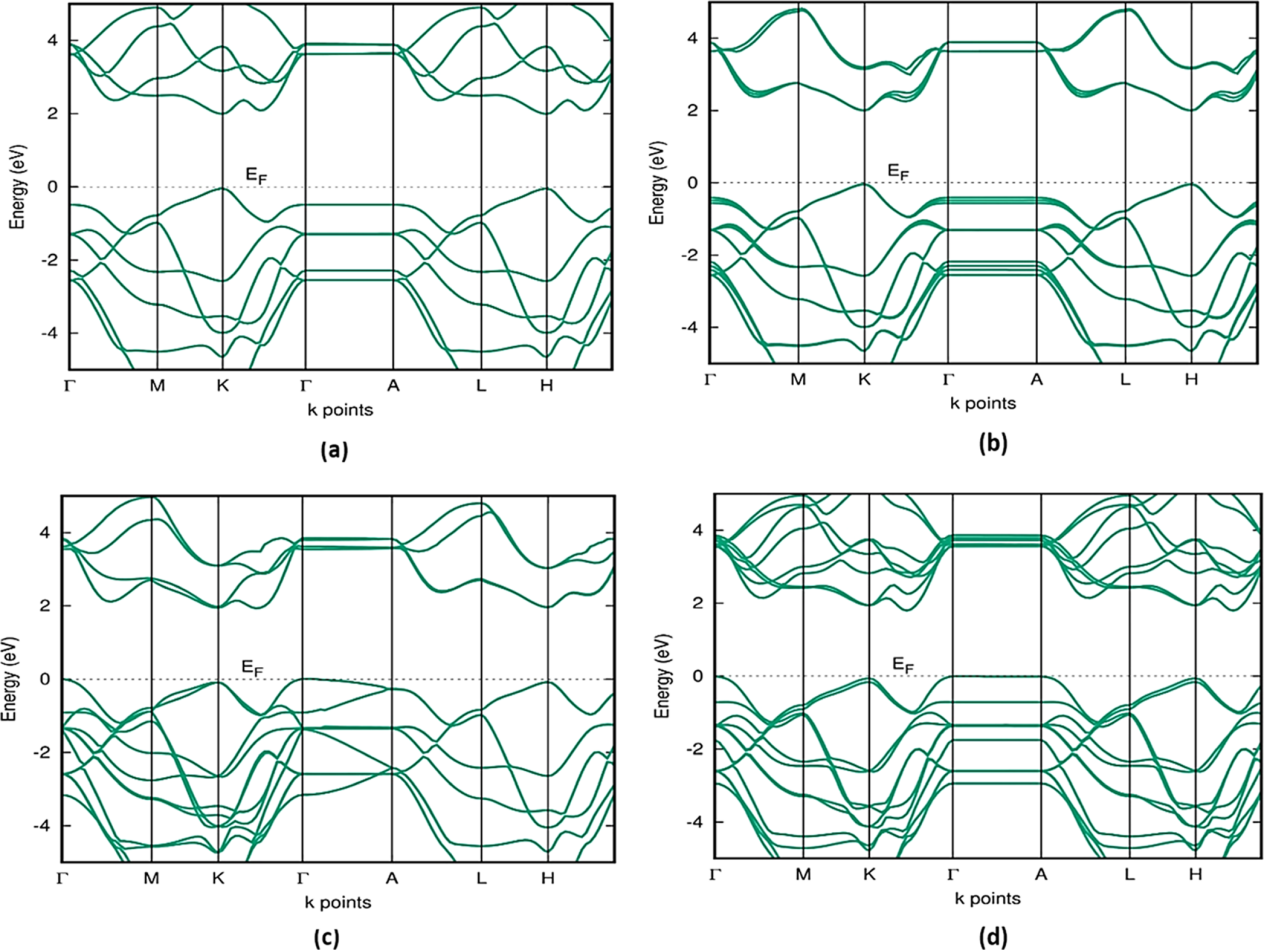
HSE06 band structure for group A (a) 1H-MoSe_2_ and (b) 3H_a_-MoSe_2_ with a direct band gap and (c) 2H-MoSe_2_ and (d) 2T-MoSe_2_ with indirect band gap. The band structure of other polymorphs in group A is given in the Supporting Information. The Fermi level is set at zero energy and marked as *E*_F_.

The conduction band minimum and valence band maximum of 1H-MoSe_2_ and 3H_a_-MoSe_2_ polymorphs occur at the same symmetric point *k* in Figure 3a,b, indicating a direct band gap with a band gap value of 2 eV.^26,27^ Because of all of the conduction band minima along the *K*–Γ path and valence band maxima positioned at the Γ point, the 2H-MoSe_2_, 2T-MoSe_2_, 4T-MoSe_2_, 3H_b_-MoSe_2_, and 2R_1_-MoSe_2_ polymorphs in group A have an indirect band gap. Figure 3c,d shows 2H-MoSe_2_ and 2T-MoSe_2_ with an indirect band gap, and other polymorphs of group A are given in Figure S1 of the Supporting Information on Page S3.^28^ The band gap value of 1.6 eV of 4T-MoSe_2_ is the same as the band gap value of the 3H_b_-MoSe_2_ polymorph. All group A polymorphs displayed similar band topologies, but the number of bands increased as the number of layers increased; this shows that all polymorphs could easily transition into one another. The band gap value and band gap type of group A are given in Table S2 of the Supporting Information on Page S4. All of the polymorphs have band degeneracy, but 4T-MoSe_2_ polymorphs have the highest, resulting in a larger effective density of state. 2H-MoSe_2_ polymorphs show a nearer value of the 1.58 eV band gap value, which is reported early by Tang et al.^29^ The band gap value of 2H-MoSe_2_ is well suited to the theoretically determined band gap value of 1.88 eV reported by Gupta et al.^13^ Additionally, the experimental value of the band gap (1.48 eV) is compared with our 2H-MoSe_2_ polymorphs that are already reported by Mahatha Patel et al.^30^ The valence band maximum and conduction band minimum are formed due to the Mo-4d and Se-3p states. Group A’s band gap range makes it appropriate for photovoltaic solar cells, photocatalysis, and water-splitting applications.

**Figure 4:**
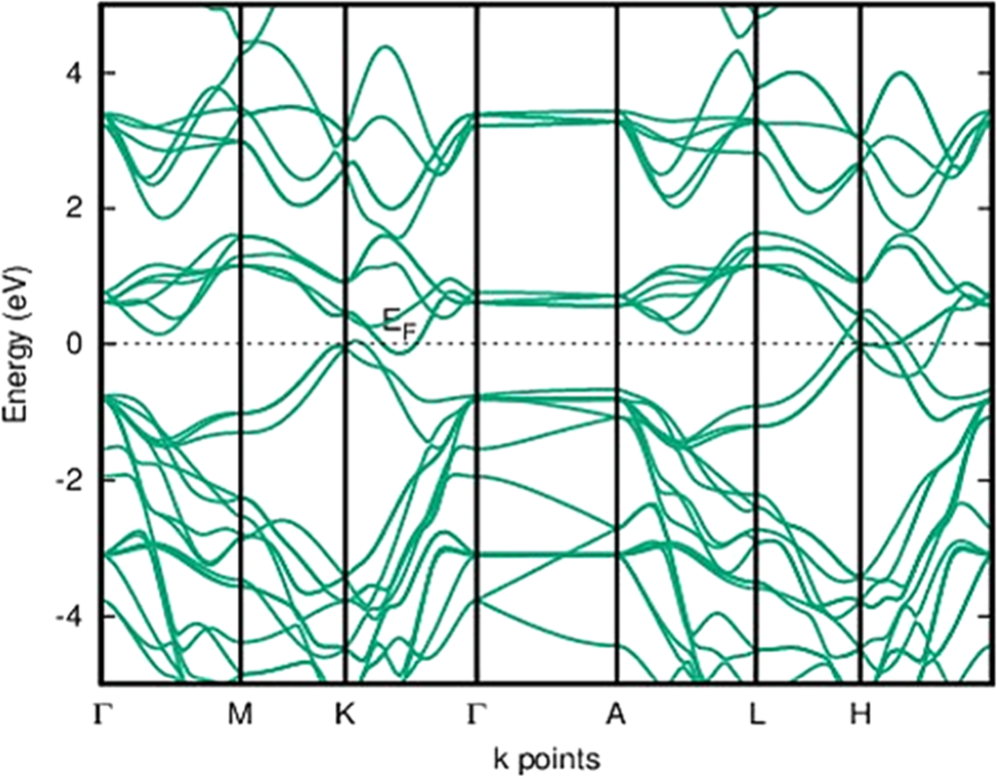
HSE06 band structure of 3T-MoSe_2_ showing a metallic appearance. The band structure of other polymorphs of group B is given in Figure S2 of the Supporting Information.

Group B polymorphs have a metallic appearance due to the overlap of the conduction and valence bands in the band structure.^27^Figure 4 shows the 3T-MoSe_2_ band structure, and the other band structure of group B is given in Figure S2 of the Supporting Information on Page S4. Although all of the bands in group B had the same structure, the number of bands in 3T-MoSe_2_ increased due to the increased layer thickness.

Surprisingly, all of the band structures in groups A and B have a flat band between Γ and A. Because of the diminishing overlap between atomic wave functions, very confined orbitals or big unit cells with well-spaced atoms can readily give birth to common flat atomic bands (FABs). In multilayer and heavy fermion systems, FABs are typical. The flatness of the band in the band structure is determined due to the interlayer spacing of the MoSe_2_.^31−33^

### 2.3 Bonding Nature

**Figure 5:**
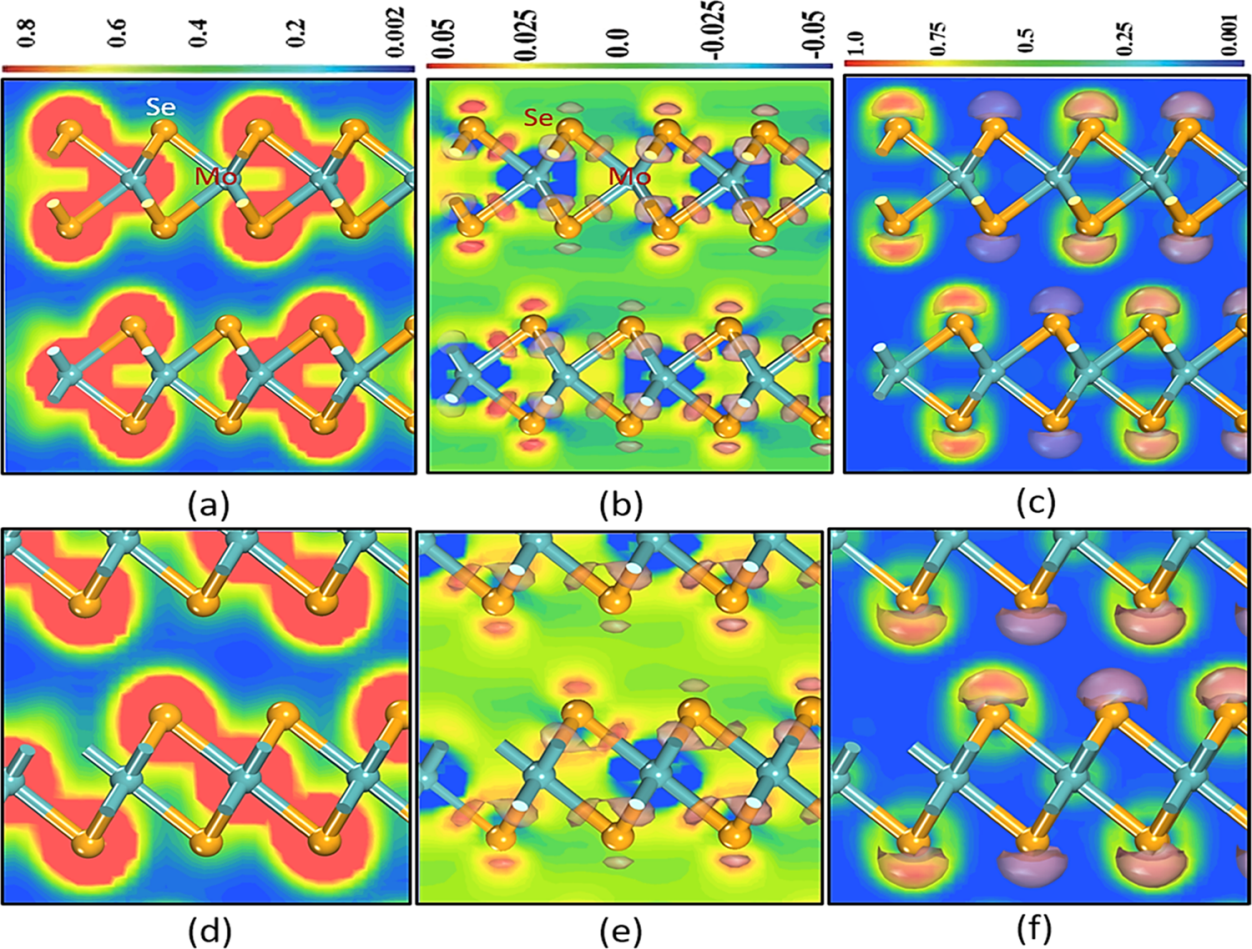
(a, d) Charge density, (b, e) charge transfer, and (c, f) ELF for hexagonal (H) and trigonal polymorph (T), respectively.

The projected valence-charge-density distribution is used to describe bonding interactions in polymorphs.^18,25^ Because of the similar nature of all of the polymorphs, the charge density, charge transfer, and electron localization function (ELF) plots of the lowest energy of hexagonal and trigonal polymorphs are the same, as shown in Figure 5.

The Se–Mo–Se bonds are trigonally connected, as shown by the hexagonal polymorph’s charge density in Figure 5a, demonstrating a normal covalent connection. Figure 5d shows a cross-shaped connection between the Se–Mo–Se trigonal polymorphs and conventional covalent bonding.^34^ The red color indicates that the cation–cation charge transfer is tightly constrained, whereas the yellow color indicates that the anion has a more diffuse location.^35^ These polymorphs have a covalent bond nature, as shown by the fact that both the cations and the anion share electrons (see Figure 5b,e).

Electron localization occurs between Se (like a cap) and Mo atoms in hexagonal and trigonal polymorph, as shown in Figure 5c,f, respectively, and is concentrated with an ELF value greater than 0.5, implying the formation of covalent bonding between Mo and Se.^36−38^ Covalent bonding is apparent in all polymorphs.

### 2.4 Vibrational Study

**Figure 6:**
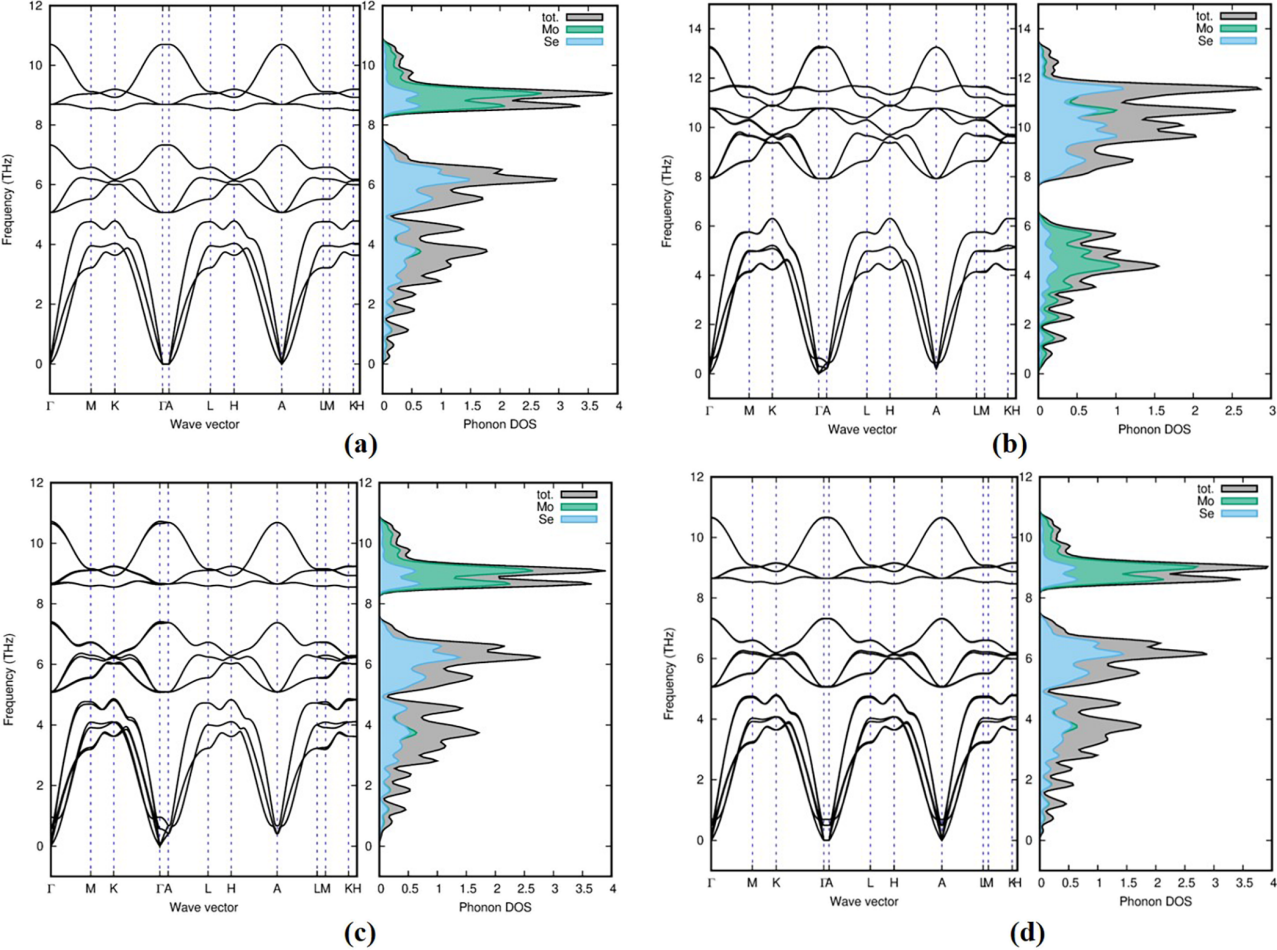
Positive phonon dispersion and phonon density of states for (a) 1H-MoSe_2_, (b) 2H-MoSe_2_, (c) 3H_b_-MoSe_2_, and (d) 2T_1_-MoSe_2_ polymorphs in group A.

The phonon is calculated for the investigated polymorphs to determine their dynamical stability, as well as the total PhDOS and phonon dispersion curves at the equilibrium volume, with the high symmetry direction of the Brillouin zone.^39^ Because of the increased forces in the polymorphs, an atom in the polymorphs is displaced from its equilibrium position, and evaluating the force associated with the system reveals the phonon frequency for a set of displacements.^40^ In general, all phonons have a genuine and positive frequency; here, six of the seven polymorphs in group A (1H-MoSe_2_, 2H-MoSe_2_, 2T-MoSe_2_, 4T-MoSe_2_, and 3H_b_-MoSe_2_) show positive mode in our investigation, indicating that they are dynamically stable. The other group A polymorph 2R_1_-MoSe_2_ exhibits a negative/soft mode of frequencies or a negative eigenvalue, suggesting that they are dynamically unstable.^41^Figure 6 shows positive phonon dispersion and phonon density of states for 1H-MoSe_2_, 2H-MoSe_2_, 3H_b_-MoSe_2_, and 2T_1_-MoSe_2_ polymorphs. Phonon dispersion and phonon density of states of the other polymorphs in group A are given in Figure S3 of the Supporting Information on Page S5.

Phonon dispersions of dynamically stable polymorphs are divided into two branches: optical (upper branch) and acoustical (lower branch). Atoms in the optical branch move in different directions, but atoms in the acoustic branch displace in the same direction with the same amplitude and phase. Relating it to other polymorphs, 2H-MoSe_2_ owned a high-frequency range of acoustic and optical modes in the region of 6 and 13 THz, respectively. Surprisingly, as shown in Figure 6, the wave vectors in phonon dispersion of the 1H-MoSe_2_, 3H_b_-MoSe_2_, and 2T-MoSe_2_ polymorphs in group A were comparable, proving that temperature and pressure may influence the transition from one phase to the other. This suggests that these polymorphs are well-suited for optical characteristics.

The stable polymorphs including the 3H_b_-MoSe_2_ polymorph have a small variation in the Γ point. The larger Mo atom dominates the higher frequencies (above 8 THz) and the smaller atom, Se, dominates the lower frequencies in group A, while in 2H-MoSe_2_ and 4T-MoSe_2_, the heavier Mo atom dominates the lower frequencies and the Se atom dominates reciprocally. Se atoms dominate the higher frequencies in group B polymorphs, while Mo atoms dominate the lower frequencies.

**Figure 7:**
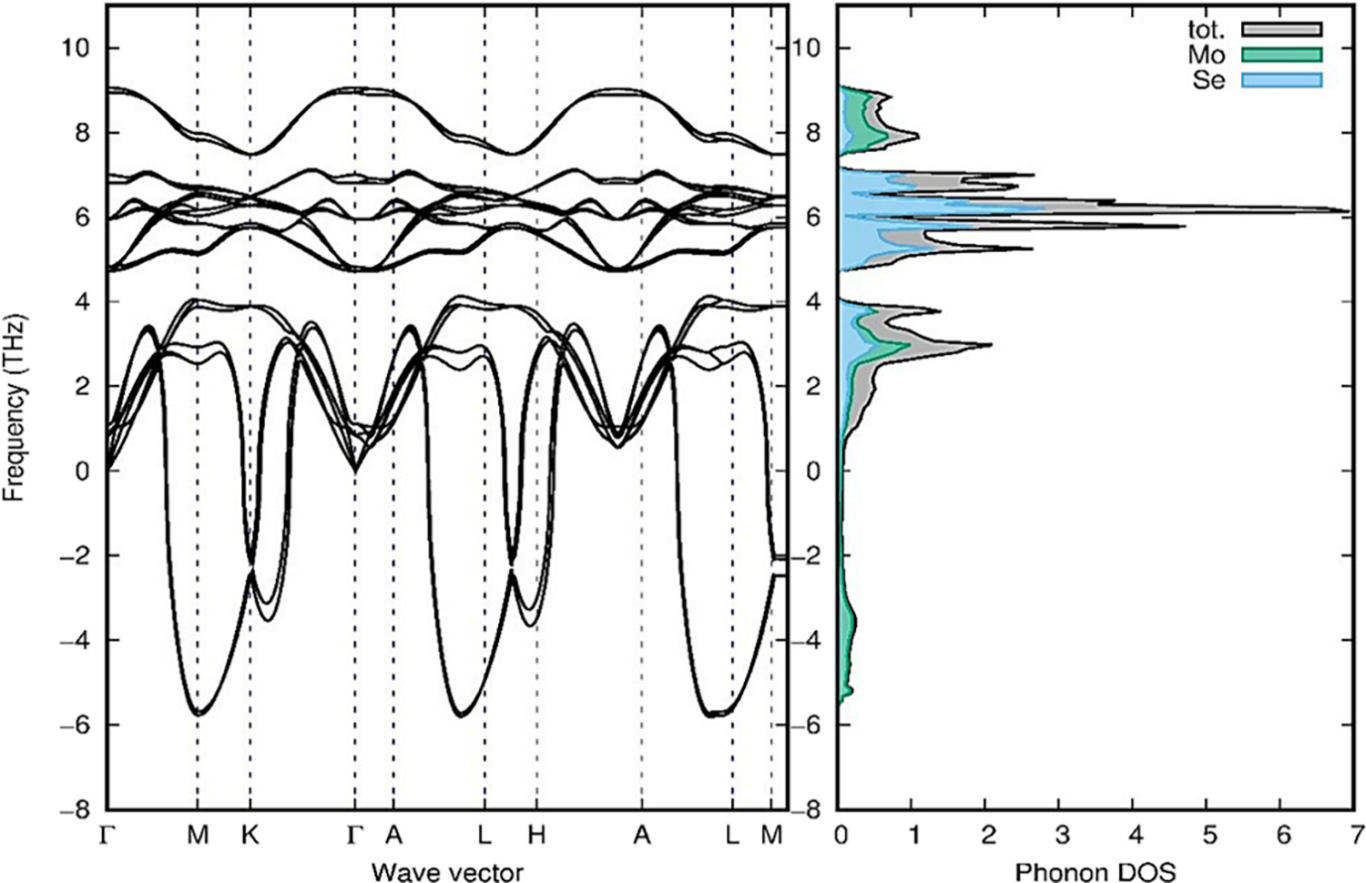
Phonon dispersion and phonon density of states for the 3T-MoSe_2_ polymorph in group B. Phonon dispersion and phonon density of states for other polymorphs are given in Figure S4 of the Supporting Information. All of the polymorphs in group B hold negative frequencies, which means that they are dynamically unstable.

The stability of polymorphs changes as a result of a decrease in the potential energy near the equilibrium atomic position. All group B polymorphs include soft/negative modes of frequency, showing unstable dynamical properties for 1T_1_-MoSe_2_, 1T_2_-MoSe_2_, 3T-MoSe_2_, and 2R_2_-MoSe_2_ polymorphs. As a result, in nature, these polymorphs are less stable. Figure 7 shows the imaginary phonon dispersion and phonon density of state for 3T-MoSe_2_ polymorphs. The other phonon dispersion and phonon density of states for group B are given in Figure S4 of the Supporting Information on Page S6.

### 2.5 Mechanical Stability

To get a better understanding of the mechanical stability of the MoSe_2_ polymorph the single-crystal elastic constant was found.^42,43^ It helps us in finding a certain deformation for the given force. The strain must be applied to support a prescribed deformation. Each stress and strain have three tensile and three shear components. This 6 × 6 symmetric matrix, which comprises 27 independent components, describes the elastic constant of the crystal. The number of components can be automatically reduced by selecting polymorphs with established symmetry. The bulk modulus, Poisson’s coefficient, and Lame constant may all be calculated using the stiffness matrix *C_ij_*. There are six elastic constants for hexagonal and trigonal polymorphs: *C*_11_, *C*_12_, *C*_13_, *C*_14_, *C*_33_, *C*_44_, *C*_55_, and *C*_66_. Theoretically, only a few polymorphs, such as 1H-MoSe_2_, 2H-MoSe_2_, and 1T-MoSe_2_, are studied in terms of their mechanical properties. Both 2H-MoSe_2_ and 2T-MoSe_2_ suggest that an elastic constant is more precisely determined than in earlier studies.^39^ This study for the first time investigated the mechanical stability of several MoSe_2_ polymorphs.

The stability criteria of elastic constant for hexagonal polymorphs are^44^
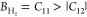
1
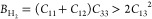
2
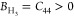
3
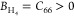
4The stability criteria of elastic constant for trigonal polymorphs are^44^
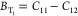
5
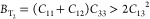
6
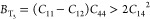
7
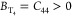
8

**Figure 8:**
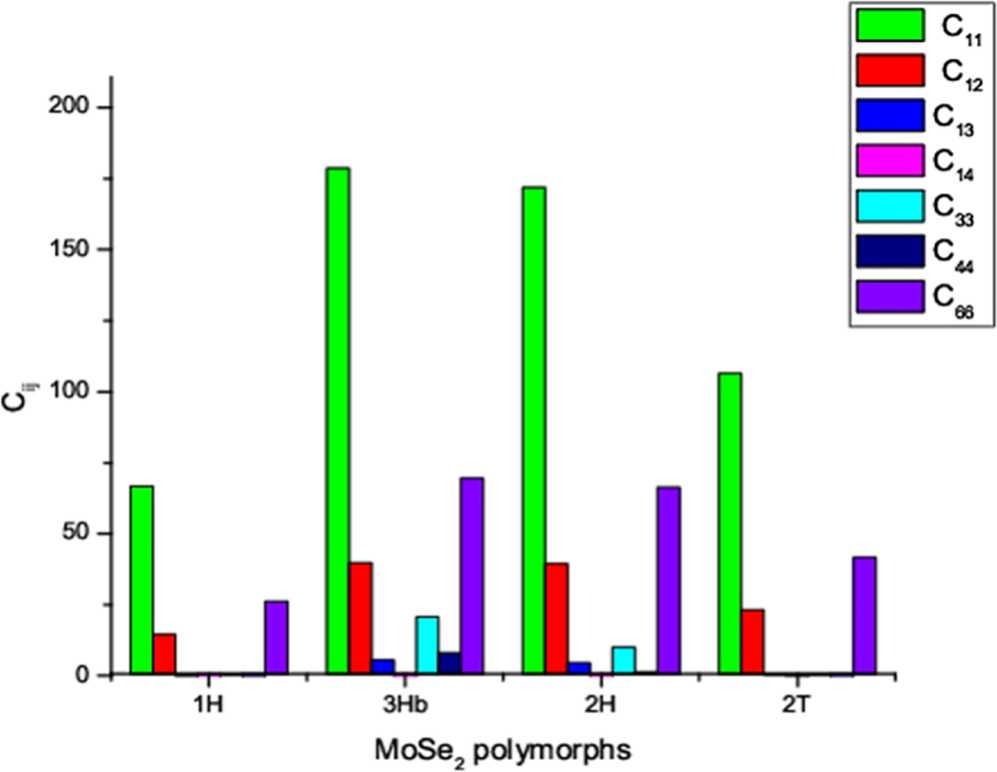
Elastic constant values for the mechanically and dynamically stable polymorphs.

Except for 3H_a_-MoSe_2_ and 4T_1_-MoSe_2_, all polymorphs in group A are mechanically stable and meet the Born stability requirement (Table 2).^5^ In group A, the 1R_1_-MoSe_2_ polymorph is mechanically stable but dynamically unstable; hence, it cannot be synthesized in the experiment. The phonon of the 4T-MoSe_2_ and 3H_a_-MoSe_2_ polymorphs are stable, but due to their unstable mechanical qualities, these polymorphs are categorized as metastable polymorphs. Even though mechanically stable, all of the group B polymorphs are unstable due to phonon instability. Because of their mechanical and dynamic stability, 4 of the 11 polymorphs are considered stable polymorphs. Elastic constants for dynamically and mechanically stable structures are plotted as a graph for a better understanding of mechanical properties (see Figure 8). From the calculation, we found that the 3H_b_-MoSe_2_ polymorph has the highest *C*_11_ value, while the 1H-MoSe_2_ polymorph has the lowest, which is clearly shown in Figure 8. In all polymorphs, the *C*_11_ value is larger than the *C*_33_ value, suggesting that the *x*/*y* direction is stiffer than the *z*-direction. *C*_12_ is greater than *C*_13_ in all stable hexagonal arrangements. This implies that tension is distributed more along the *x*- and *z*-axes and less along the *y*-axis. Because of the low *C*_44_ value, shear deformation is easily accomplished in all stable polymorphs.

**Table 2:** Calculated Single-Crystal Elastic Constants *C_ij_* (in GPa) and Born Criteria for All of the Polymorphs

polymorph	1H-MoSe_2_	3H_a_-MoSe_2_	3H_b_-MoSe_2_	2H-MoSe_2_	4T-MoSe_2_	2T-MoSe_2_	2R_1_-MoSe_2_	1T_1_-MoSe_2_	1T_2_-MoSe_2_	3T-MoSe_2_	2R_2_-MoSe_2_
crystal system	hexagonal	hexagonal	hexagonal	hexagonal	trigonal	trigonal	rhombohedral	trigonal	trigonal	trigonal	rhombohedral
*C*_11_	66.72	100.06	178.67	171.98(131)^45^	–26 527.6	106.39	182.81	212.38	112.46	195.09	180.51
*C*_12_	14.54	22.37	39.70	39.38(39)^45^	–26 571.5	23.24	42.36	16.05	–78.19	9.03	5.41
*C*_13_	0.04	16.23	5.50	4.49	12 985.6	0.13	9.76	23.02	3.55	16.05	23.67
*C*_14_	0	0	0	0	0.96	0.07	2.88	0.00	0.00	3.25	4.531
*C*_33_	0.09	–0.48	20.62	9.94	0.75	0.13	23.16	59.61	44.89	52.84	30.34
*C*_44_	0.01	0.03	8.00	1.32	0.70	0.003	11.97	13.40	62.13	28.51	7.86
*C*_66_	26.09	38.84	69.48	66.30	21.94	41.56	70.22	98.16	95.32	93.03	87.55
born	yes	no	yes	yes	no	yes	yes	yes	yes	yes	yes

The Voigt (V), Reuss (R), and Hill (H) moduli over the elastic stiffness moduli are computed for all of the polymorphs by applying mechanical force. Table 3 shows the Voigt (V), Reuss (R), and Hill (H) moduli over the elastic stiffness moduli of the stable polymorphs. Additionally, averaging the single-crystal elastic constant leads to finding bulk modulus *B* and shear modulus *G*.^46^

**Table 3:** Calculated Bulk Modulus *B* (in GPa), Shear Modulus *G* (in GPa), Poisson’s Ratio (ν), Young’s Modulus *E* (in GPa), and Pugh Ratio (*G*/*B*)^a^

polymorph	*B*_V_	*B*_R_	*B*_H_	*G*_V_	*G*_R_	*G*_H_	ν_v_	ν_R_	ν_H_	*E*_V_	*E*_R_	*E*_H_	Pugh ratio
1H-MoSe_2_	18.09	0.09	9.09	13.15	0.042	6.597	0.20	0.30	0.20	31.75	0.11	15.93	0.72
3H_b_-MoSe_2_	53.26	18.69	35.98	38.91	14.26	26.59	0.20	0.19	0.20	93.88	34.11	64.00	0.73
2H-MoSe_2_	50.07	9.66	29.86	34.15	2.95	18.55	0.22	0.36	0.24	83.48	8.04	46.12	0.62
2T-MoSe_2_	28.89	0.25	14.57	20.95	0.007	10.48	0.20	0.48	0.21	50.62	0.02	25.36	0.71

a Subscript V shows the Voigt bound, R shows the Reuss bound, and H shows the Hill bound.

In Voigt approximation, bulk *B* and shear *G* can be defined as
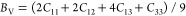
9
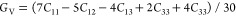
10

And the Reuss approximation of bulk and shear is defined as
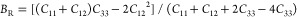
11
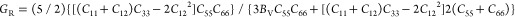
12

The Hill approximation is used widely for the effective result of the bulk modulus (*B*) and shear modulus (*G*), which can be expressed as
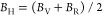
13
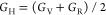
14

Further, on the basis of the bulk modulus (*B*) and shear modulus (*G*), Pugh ratio (*G*/*B*), Poisson’s ratio (ν), and Young’s modulus (*E*) are obtained using the following expression
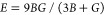
15
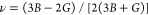
16

Table 3 shows the findings of the elastic constant. Consequently, all of the stable polymorphs had *B* > *G*, showing that mechanical stability is restricted by shear modulus. The bulk modulus (*B*) and shear modulus (*G*) can be used to predict the hardness of polymorphs. In the current study, the 3H_b_-MoSe_2_ polymorph has higher bulk modulus and shear modulus values (see Table 3) These results reveal that the 3H_b_-MoSe_2_ polymorph has higher hardness.

Another significant aspect of mechanical characteristics is the ability to distinguish between ductile and brittle behavior in stable polymorphs. Pugh’s ratio (ratio of the shear modulus to the bulk modulus (*G*/*B*)) and Poisson’s ratio (ν) are used to figure out the brittle/ductile behavior of polymorphs. The key value of Pugh’s ratio was 0.5, which allowed us to discriminate between ductile and brittle materials. If the material is more than (less than) 0.5, it will be brittle (ductile).^14,43^ According to Pugh’s ratio, all stable polymorphs have a value greater than 0.5, suggesting that all polymorphs were brittle.

Using Poisson’s ratio (ν), we can distinguish between brittle and ductile materials. The Poisson’s ratio (ν) for ductile materials should be larger than 0.26.^43^ All stable polymorphs are fragile in nature since their ν values are smaller than 0.26.^43^ Among stable polymorphs, the 2H-MoSe_2_ polymorph has a higher Poisson’s ratio (0.243). The 1H-MoSe_2_ and 3H_b_-MoSe_2_ Poisson’s ratios (ν) are most obvious in the concrete range. The Poisson’s ratio (ν) of the 2H-MoSe_2_ polymorph is found in the cast iron range.

The fact that Young’s modulus (*E*) has a positive value suggests that the atoms in stable polymorphs are compressible. In this study, Young’s modulus (*E*) is lower for 1H-MoSe_2_ and higher for 3H_b_-MoSe_2_ polymorphs, implying that the atoms in 3H_b_-MoSe_2_ polymorphs are more compressible than those in other polymorphs. The elastic stiffness moduli, Poisson’s ratio (ν), Young’s modulus (*E*), and Pugh’s ratio of unstable polymorphs are given in Table S3 in the Supporting Information on Page S6

### 2.6 Anisotropic

Elastic anisotropic are investigated for stable polymorphs. To understand the elastic anisotropic, shear anisotropic (*A*_G_), anisotropic compressibility (*A*_B_), and universal anisotropic (*A*^u^) (see Table 4) are calculated for the mechanically and dynamically stable polymorphs.^47^

**Table 4:** Calculated Shear Anisotropic *A*_G_, Anisotropic Compressibility *A*_B_, and Universal Anisotropic *A*^U^ for the Stable Polymorphs

polymorphs	*A*_G_	*A*_B_	*A*^U^
1H-MoSe_2_	1354.6	1882.23	∞
2H-MoSe_2_	50.22	37.00	56.94
2T-MoSe_2_	1400.75	1049.92	∞
3H_b_-MoSe_2_	8.679	13.72	10.490

The elastic anisotropic for compressibility and shear is expressed as
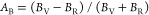
17
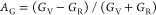
18And universal anisotropic can be expressed as
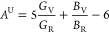
19

**Figure 9:**
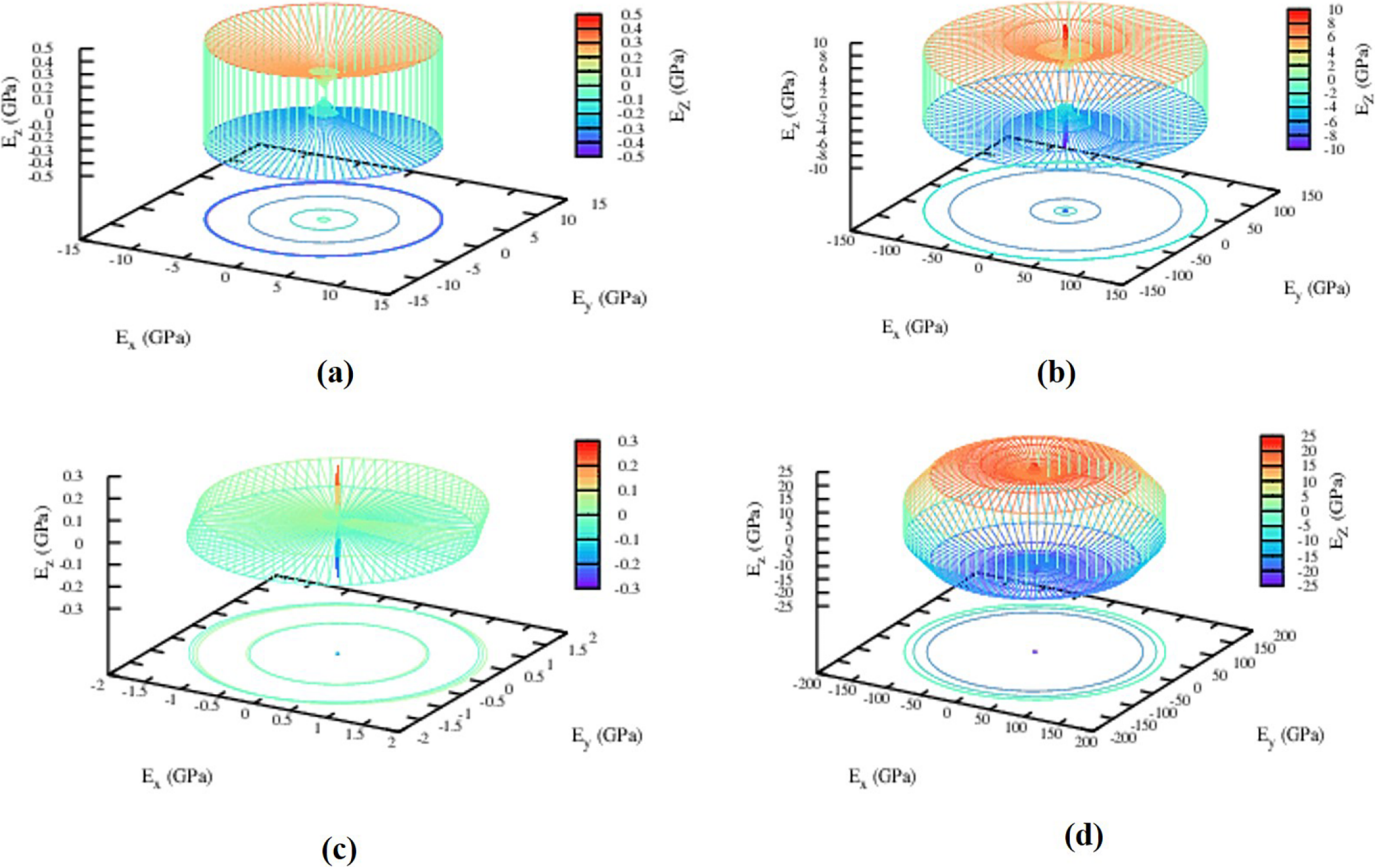
3D-plot of Young’s modulus (*E*) for stable polymorphs: (a) 1H-MoSe_2_, (b) 2H-MoSe_2_, (c) 2T-MoSe_2_, and (d) 3H_b_-MoSe_2_.

For isotropic materials, the value of *A*^U^ is zero, but a high value of *A*^U^ shows the presence of elastic anisotropy. The physical quantities and orientation of Young’s modulus (*E*) and Poisson’s ratio (ν) can be used to show that polymorphs have isotropic characteristics. Young’s modulus (*E*) determines the polymorph’s orientation, which is derived using the given elastic compliance constants.^46^ Young’s modulus (*E*) surface should be perfectly spherical for isotropic materials. Figure 9 shows a three-dimensional (3D) plot of Young’s modulus (*E*) for dynamically stable polymorphs. Young’s modulus (*E*) is the highest for 3H_b_-MoSe_2_, and the lowest Young’s modulus (*E*) is obtained for 1H-MoSe_2_.^43,47^

### 2.7 Thermodynamical Properties

**Figure 10:**
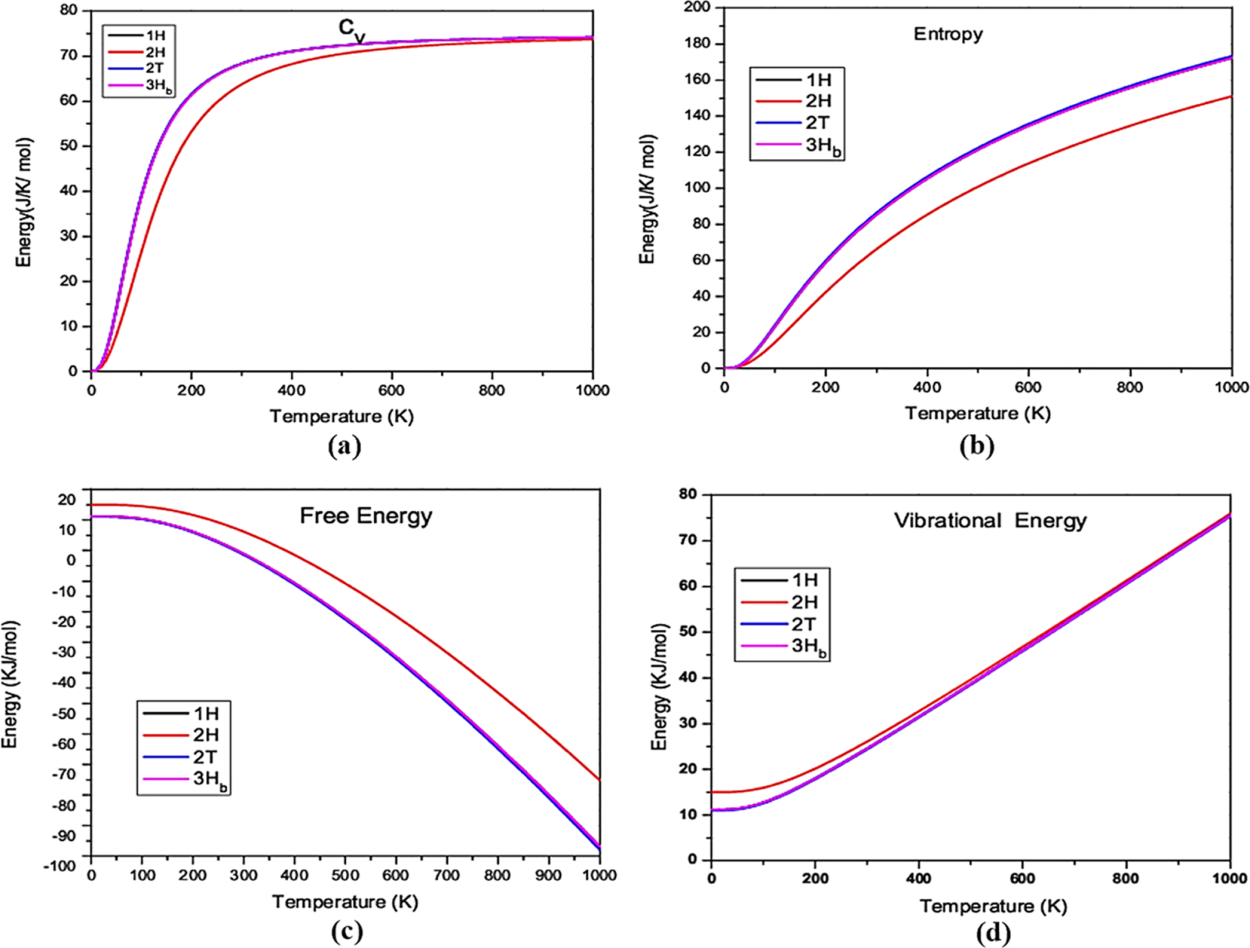
Temperature as a function of specific heat at (a) constant volume “*C*_v_”, (b) entropy “*S*”, (c) internal energy “*E*”, and (d) vibrational energy “*F*” for dynamically stable polymorphs.

The effect of phonon on dynamically as well as mechanically stable structures has been examined through thermodynamic behavior (1H-MoSe_2_, 2H-MoSe_2_, 3H_b_-MoSe_2_, and 2T-MoSe_2_). For the stable polymorphs, temperature-dependent thermodynamic functions such as specific heat at constant volume “*C*_v_”, entropy “*S*”, internal energy “E”, and vibrational free energy “*F*” is computed.^32^Figure 10 demonstrates how vibrational energy increases and free energy decreases exponentially, following Debye’s law. Compared to the other stable polymorphs, the 2H-MoSe_2_ polymorph shows high vibrational energy, and the free energy is high for the 2H-MoSe_2_ polymorph. At extremely low temperatures, entropy stays constant for all polymorphs, and at an absolute zero, it becomes zero.^46^ At 1000 K, 2T-MoSe_2_, 3H_b_-MoSe_2_, and 1H-MoSe_2_ polymorphs obtained the constant value 170 J K^–1^ mol^–1^, but 1H-MoSe_2_ polymorph showed 140 J K^–1^ mol^–1^ of energy as a function of temperature. All of the stable polymorphs follow the third rule of thermodynamics by increasing entropy as the temperature rises.

At constant volume, specific heat *C*_v_ increases linearly with temperature, implying that all polymorphs follow Debye’s T^3^ rule for constant volume and the conventional Dulong–Petit law for elevated temperatures. 1H-MoSe_2_, 2T-MoSe_2_, and 3H_b_-MoSe_2_ have the same *C*_v_.^39^ Our data for various temperatures obeys the two important laws of thermodynamics, implying that all examined polymorphs have thermodynamic stability even for greater pressure ranges. Although there have not been any findings, either theoretical or experimental, it is difficult to compare the outcomes.

### 2.8 IR and Raman Spectra

#### 2.8.1 IR Spectra

**Figure 11:**
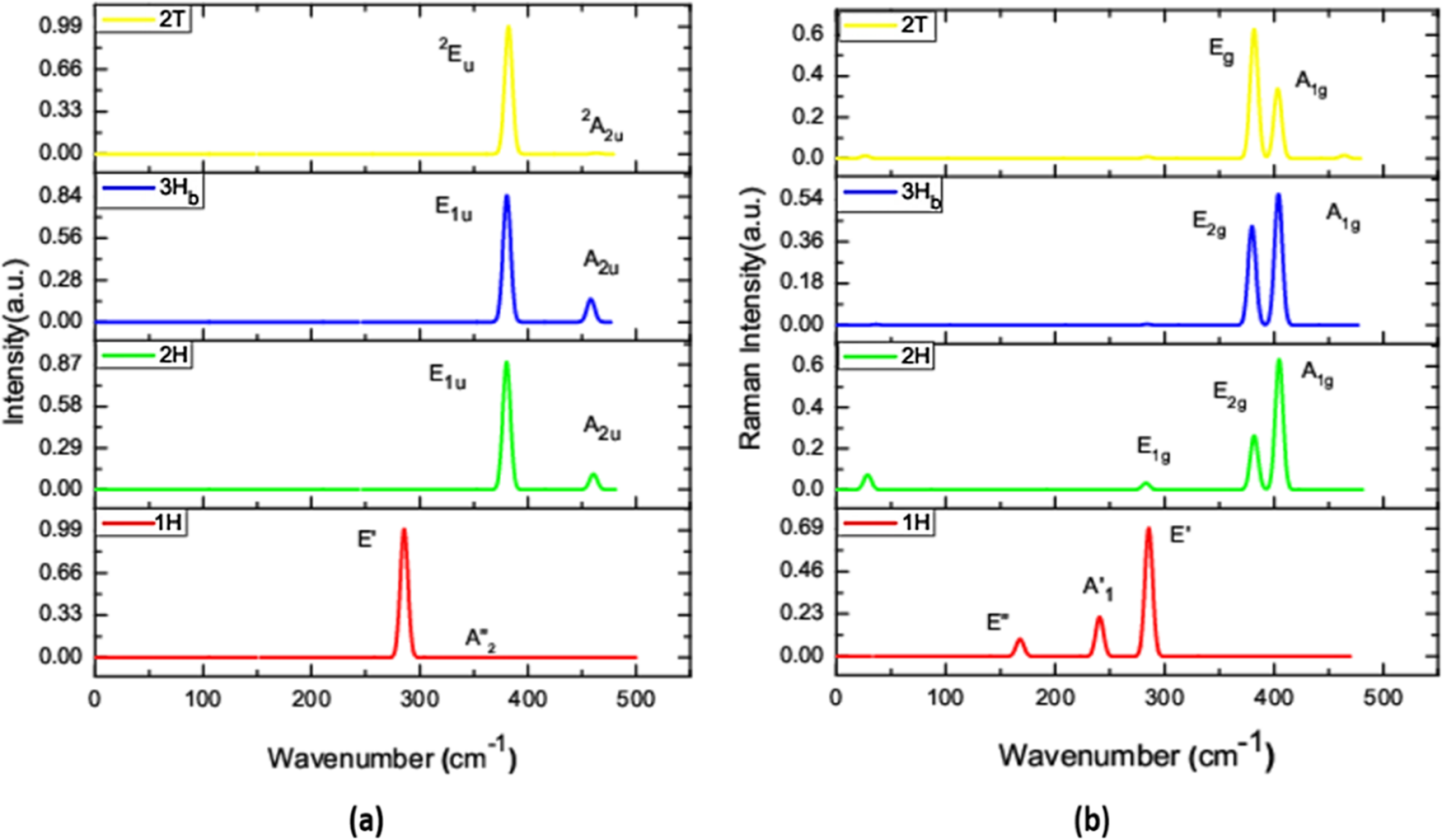
(a) IR intensity as a function of wavenumber and (b) Raman intensity as a function of wavenumber for dynamically stable polymorphs.

IR spectra are studied for the stable polymorphs, and the corresponding modes of representation are evaluated in this paper.^46^ Because of the Se–Mo–Se vibrations, modes of vibrations are prominent in high-frequency regions and some modes are found in low frequencies regions because of Mo–Se vibration (see Table 5).^43^ Surprisingly, all of the polymorphs are symmetric to the principal axis, and it is double degenerate, which is two-dimensional irreducible representations. Specifically, 1H-MoSe_2_ polymorphs are antisymmetric for the reflection into the horizontal plane, and it shows A″_2_ and E′ modes, which confirms that it is a single-layer material. From the crystal symmetric 2H-MoSe_2_, 3H_b_-MoSe_2_ polymorphs displace in the bulk material modes A_2u_ and E_1u_. 2T-MoSe_2_ is active in A_2u_ and E_u_ modes, which shows a double layer. Not to be surprised, 2H-MoSe_2_ and 3H_b_-MoSe_2_ have the same IR active modes, which show the same symmetric operators. Compared to the other polymorphs, 1H-MoSe_2_ projects high frequency, as well as 2T-MoSe_2_ from Figure 11 shows a considerable number of IR active modes.

**Table 5:** Raman Active Modes and IR Active Modes for the Dynamically Stable Polymorphs

polymorph	Raman active mode (cm^–1^)	IR active mode (cm^–1^)
1H-MoSe_2_	E′: 285	E′: 285
	E″: 167	A″_2_: 350
	A′_1_: 240	
2H-MoSe_2_	^2^E_2g_: 28, 381	A_2u_: 460
	^2^E_1g_: 282	E_1u_: 380
	^1^A_1g_: 404	
3H_b_-MoSe_2_	^2^E_2g_: 36, 379	A_2u_: 458
	^2^E_1g_: 284	E_1u_: 380
	A_1g_: 404	
2T-MoSe_2_	^2^E_g_: 26, 284, 381	^2^E_u_: 283, 382
	^2^A_1g_: 41, 403, 464	^2^A_2u_: 401, 463

#### 2.8.2 Raman Spectra

Studied polymorphs show the signature Raman active modes, as shown in Figure 11. In our study, we noted that out-of-plane A_1g_ mode dominates in 3H_b_-MoSe_2_ and in 2H-MoSe_2_ polymorphs, which show single degenerate wave functions.^48,49^ While the 2T-MoSe_2_ polymorph is dominated by the in-plane E_g_ mode. We see that modes of polymorphs are red-shifted from approximately 40 cm^–1^ when compared to the modes of 1H-MoSe_2_. The observed red shift is caused by larger interlayer distances, which lead to an increase in the dielectric screening of the long-range Coulomb forces and thus overall restoring force gets reduced on the atom. 2H-MoSe_2_ and 3H_b_-MoSe_2_ polymorphs in group A display Raman-active modes E_1g_ and A_1g_, as shown in Figure 11b. The polymorph 2H-MoSe_2_ is double degenerate, indicating that it is bulk material. 2T-MoSe_2_ polymorphs show many Raman-active modes and the highest mode of peak (Figure 11) compared to other polymorphs.^49^ We noted that Raman’s active mode of the 2H-MoSe_2_ polymorph is the same as the earlier reported value. But for the remaining polymorphs, there is still a lack of other theoretical IR studies in the literature on these polymorphs. This makes it difficult to verify this result due to a lack of literature data.

## 3 Conclusions

Foremost 11 different MoSe_2_ polymorphs are put forward and studied using total-energy calculations, band structure analysis, phonon density of states, and elastic constants calculations in DFT. Our detailed study shows that polymorphs in group A have minimum energy, compared to group B polymorphs. The minimum energy of group A polymorphs is very close, although with a varying range of volume. Polymorphs in group A are semiconductors with direct and indirect band gaps. 1H-MoSe_2_ and 3H_a_-MoSe_2_ in group A show a direct band gap at 2 eV, and the remaining polymorphs in group A show an indirect band gap within the range of 1.6–1.8 eV. All polymorphs in group B are dynamically unstable. In group A, 1H-MoSe_2_, 2H-MoSe_2_, 2T-MoSe_2_, and 3H_b_-MoSe_2_ polymorphs are mechanically, dynamically, and thermodynamically stable. The metastable state is depicted for the 4T-MoSe_2_ and 3H_a_-MoSe_2_ polymorphs because of their unstable mechanical properties and stable phonon properties. When compared to other polymorphs, the 3H_b_-MoSe_2_ polymorph in particular exhibits greater hardness. The thermal efficiency was highest for the 2H-MoSe_2_ polymorph. It is concluded that four of the 11 polymorphs in group A will adhere to the stable criterion since they are simple to synthesize and suitable for viable applications like photocatalytic and photovoltaic.

## 4 Methodology

The VASP code is enforced for all calculations within the periodic density functional theory.^20,50^ The projector-augmented wave (PAW) method is used to describe the interaction of core and valence electrons.^51−53^ Initially, the structure of all of the polymorphs is optimized with the help of the Perdew–Burke–Ernzerhof (PBE) exchange–correlation functional.^54^ The DFT/vdw-df2 method is used to obtain the PBE-level optimized structure. The energy–volume curve was generated for the optimized structures to find their lowest energy. Only for the large e-cut, parameters of the structure can predict reliability, so we used a 550 eV energy cutoff. The screened hybrid function was used to find the electronic properties of polymorphs that are optimized at the PBE level; the screened hybrid function was proposed by Heyd, Scuseria, and Ernzerhof (HSE06).^55,56^ For the structural optimization and the electronic polymorph studies, we have used a Monkhorst–Pack 2 × 2 × 2 as *k*-mesh. Bands of the polymorphs are computed by solving the periodic Kohn–Sham equation on 10 *k*-points along each direction of high symmetry of the irreducible part of the first Brillouin zone. Charge density, charge transfer, and electron localization function (ELF) analyses were performed using the CASTEP code, and this helped to understand the bonding nature and interaction between the MoSe_2_ polymorphs. PHONOPY software is used to calculate phonon dispersion and phonon density of state for suitable supercell mode performed for the supercells of the polymorphs.^39^ The force constant on the supercell is calculated with the help of the VASP code.^51,53,57^ Every atom in the binary system is displaced by applying a finite displacement of 0.007 Å in the *x*, *y*, and *z* directions to get the matrices to form the force constant. After obtaining the force constant, we construct a dynamical matrix for different q vectors in the Brillouin zone. The dynamical matrices are solved to get the result of eigenvalues of phonon frequency and eigenvectors of phonon mode. Suitable supercell mode and Monkhorst–Pack grid is given in Table S4 of the Supporting Information on Page S7. The dynamical stability of all polymorphs is checked by remarking imaginary and real modes of polymorphs. Thermal properties are obtained for the studied polymorphs including heat capacity, free energy, and entropy of the system.^39^ In this present work, the mechanical stability of all polymorphs is understood by computing single-crystal elastic constants. Each crystal system has applied a set of strains (−0.015, −0.010, −0.005, 0.000, 0.005, 0.010, and 0.015), and the stress tensor is calculated. VASPKIT is used to evaluate the elastic constants by linear fitting of the stress–strain curve.^50^ The CASTEP package is used to obtain the Raman and IR spectra for all of the polymorphs of MoSe_2_.^49^ We used an optimized structure for the CASTEP computational code to get an accurate result.^58^

## Supporting Information Available

Minimum energy; formation energy of all of the polymorphs; electronic structure; mechanical property data in detail; phonon dispersion relation and band structure of polymorphs; and *k*-grid and supercell size of MoSe_2_ polymorphs (PDF)
