# Supplementary material for: First-Principles Insights into the Relative Stability, Physical Properties, and Chemical Properties of MoSe2
Source: ACS Omega. 2023 Apr 5;8(15):13799–812. doi: 10.1021/acsomega.2c08217 (PMC10116531; doi:10.1021/acsomega.2c08217)
Supplement: Supplementary file 1 — ao2c08217_si_001.pdf [file ao2c08217_si_001.pdf]

Supplementary Information of First-principles insights into the relative stability, physical and chemical properties of $MoSe_2$

Lathifa Banu.S[a], Vasu Veerapandy[a], Helmer Fjellvåg[b], and Ponniah Vajeeston[b*]

[a] *Department of Computational Physics, School of Physics, Madurai Kamaraj University, Madurai 625021, Palkalai Nagar, Tamil Nadu, India.*

[b] *Department of Chemistry and Center for Materials Science and Nanotechnology, University of Oslo, Oslo 0371, Norway.*

1. Structure and the stability of $MoSe_2$ polymorphs

Table S1. **The calculated minimum energy(E) with respect to the volume (Å ^{3}) of optimized $MoSe_2$ polymorphs and the formation energy of optimized $MoSe_2$ polymorphs**

Polymorph	Volume (Å ^{3})	Minimum Energy (eV/f.u.)	*(ΔH) =E_0 ($MoSe_2$)-[E_0(Mo)+E_0(Se)](eV/f.u.)
**Group-A**			
1H-$MoSe_2$	166.20	-20.04	-2.13
2H-$MoSe_2$	64.12	-20.06	-2.15
$3H_a$-$MoSe_2$	112.04	-20.05	-2.14
$3H_b$-$MoSe_2$	62.09	-20.07	-2.16
2T-$MoSe_2$	103.95	-20.07	-2.16
4T-$MoSe_2$	81.86	-20.08	-2.17
$2R_1$-$MoSe_2$	60.93	-20.08	-2.17
**Group-B**			
$1T_1$- $MoSe_2$	58.60	-19.33	-1.43
$1T_2$- $MoSe_2$	59.14	-19.41	-1.51
3T-$MoSe_2$	57.94	-19.44	-1.54
$2R_2$-$MoSe_2$	59.86	-19.40	-1.5

***(ΔH) = Formation energy, E_0($MoSe_2$) = Energy of $MoSe_2$, E_0(Mo)=Energy of Mo and E_0(Se_2) =Energy of Se_2**

2. Structural features of Mo-Se

* Corresponding author
Email address: vajeeston.ponniah@kjemi.uio.no

1H-$MoSe_2$ is Molybdenite structured and crystallizes in the trigonal *P*$\overline{6}$*m2* space group. The structure is two-dimensional and consists of two $MoSe_2$ sheets oriented in the (0, 0, 1) direction. Mo (1) $^{4+}$ is bonded to six equivalent Se (2,3) $^{2-}$ atoms to form distorted edge-sharing Mo_3Se_6 pentagonal pyramids. All Mo (1)–Se (2,3) in 1H-$MoSe_2$ had bond lengths are 2.52 Å. Se (2,3) $^{2-}$ is bonded in a 3-coordinate geometry to three equivalent Mo (1) $^{4+}$ atoms. 2H-$MoSe_2$ consists of Mo (1) $^{4+}$ is bonded to six equivalent Se (1) $^{2-}$ atoms to form distorted edge-sharing Mo_3Se_6 and form a hexagonal structure. Mo (1)–Se (1) of 2H-$MoSe_2$ had lengths are 2.52 Å. Se (1) $^{2-}$ is bonded in a 3-coordinate geometry to two equivalent Mo (1) $^{4+}$ atoms with the space group *P*6_3*/mmc*.

3H$_a$-$MoSe_2$ structure with the space group *P*$\overline{6}$*m2* shows Two equivalent Mo (1) $^{1+}$ is bonded each to six equivalent Se (1) $^{2-}$ atoms and six equivalent Se (3) $^{2-}$ and Mo (2) $^{1+}$ is bonded to six equivalent Se (2) $^{2-}$to form distorted center-sharing Mo_3Se_6 and form hexagonal structure. 3H$_a$-$MoSe_2$ of Mo (1)–Se (1) Mo (1)–Se (3) and Mo (1)–Se (2) bond lengths are 2.51 Å. Se (1) $^{2-}$ is bonded in a 3-coordinate geometry to two equivalent Mo (1) $^{1+}$and one Mo (2) $^{1+}$ atom. The structure of 2T-$MoSe_2$ with the space group *P*$_3$*m1* had two equivalent Mo (1)$^{1+}$ bonded each to six equivalent Se (1) $^{2-}$ atoms and six equivalent Se (3) $^{2-}$ to form distorted center-sharing Mo_3Se_6 and form trigonal structure. Mo (1)–Se (1) obtained the bond lengths are 2.51 Å in 2T-$MoSe_2$. Se (1) $^{2-}$ is bonded in a 3-coordinate geometry to two equivalent Mo (1) $^{1+}$ atom.

Two equivalent Mo (1)$^{1+}$ is bonded each to six equivalent Se (1) $^{2-}$ atoms and six equivalent Se (3) $^{2-}$ and Two equivalent Mo (2) $^{1+}$ is bonded each to six equivalent Se (2) $^{2-}$ atoms and six equivalent Se (4) $^{2-}$ to form distorted center-sharing Mo_4Se_8 and form trigonal structure. All Mo (1)–Se (1), Mo (1)–Se (3), Mo (2)–Se (2), and Mo (2)–Se (4) are bonded in bond lengths are 2.51 Å. Se (1) $^{2-}$ Se (2) $^{2-}$ Se (3) $^{2-}$and Se (4) $^{2-}$ are bonded in a 3-coordinate geometry to two equivalent Mo (1) $^{1+}$and two equivalent Mo (2)$^{1+}$ atoms in the structure of 4T-$MoSe_2$ with the space group *P*$\overline{6}$*m1*. 2R$_1$-$MoSe_2$ structure with the space group had *R3m* Mo (1) $^{1+}$ is bonded to three equivalent Se (1) $^{2-}$ and three equivalent Se (2) $^{2-}$ atoms to form based center-sharing Mo_3Se_6 and form rhombohedral structure. Mo (1)–Se (1,2) 2R$_1$-$MoSe_2$ had bond lengths are 2.51 Å. Se (1,2) $^{2-}$ is bonded in a 3-coordinate geometry to Mo (1) $^{1+}$ atom.

The structure of 1T$_1$-$MoSe_2$ with the space group *P3m1* and 1T$_2$-$MoSe_2$ with the space group *P*$_3$*m1* polymorphs are consisting of Mo (1) $^{1+}$ is bonded each to six equivalent Se (1) $^{2-}$ atoms and Se (1) $^{2-}$ is bonded in a 3-coordinate geometry to two equivalent Mo (1) $^{1+}$ atom to form trigonal structures in with the bond lengths 2.52 Å and 2.53 Å with respectively. 3T-$MoSe_2$ had Two equivalent Mo (1) $^{1+}$ bonded each to six equivalent Se (1) $^{2-}$ atoms and six equivalent

Se (3) $^{2-}$ and four equivalent Mo (2) $^{1+}$ had each to eight equivalent Se (2) $^{2-}$ atoms and eight equivalent Se (4) $^{2-}$ to form distorted center-sharing Mo_3Se_6 and form trigonal structure. Mo (1)–Se (3), Mo (1)–Se (6), Mo (2)–Se (2,5), and Mo (2)–Se (4,1) are bonded in bond lengths are 2.53 Å. Se (1) $^{2-}$ Se (2) $^{2-}$ Se (3) $^{2-}$and Se (4) $^{2-}$ are bonded in a 3-coordinate geometry to two equivalent Mo (1) $^{1+}$and two equivalent Mo (2) $^{1+}$ atoms in the structure of 3T-$MoSe_2$ with the space group *R3m*. 2R_2-$MoSe_2$ structure with the space group had *P3m1* Mo (1) $^{1+}$ is bonded to four equivalent Se (1) $^{2-}$ and four equivalent Se (2) $^{2-}$ atoms to form a biased center-sharing Mo_4Se_8 and form a rhombohedral structure. Mo (1)–Se (1,2) 2R_1-$MoSe_2$ had bond lengths are 2.53 Å.

3. Electronic structure

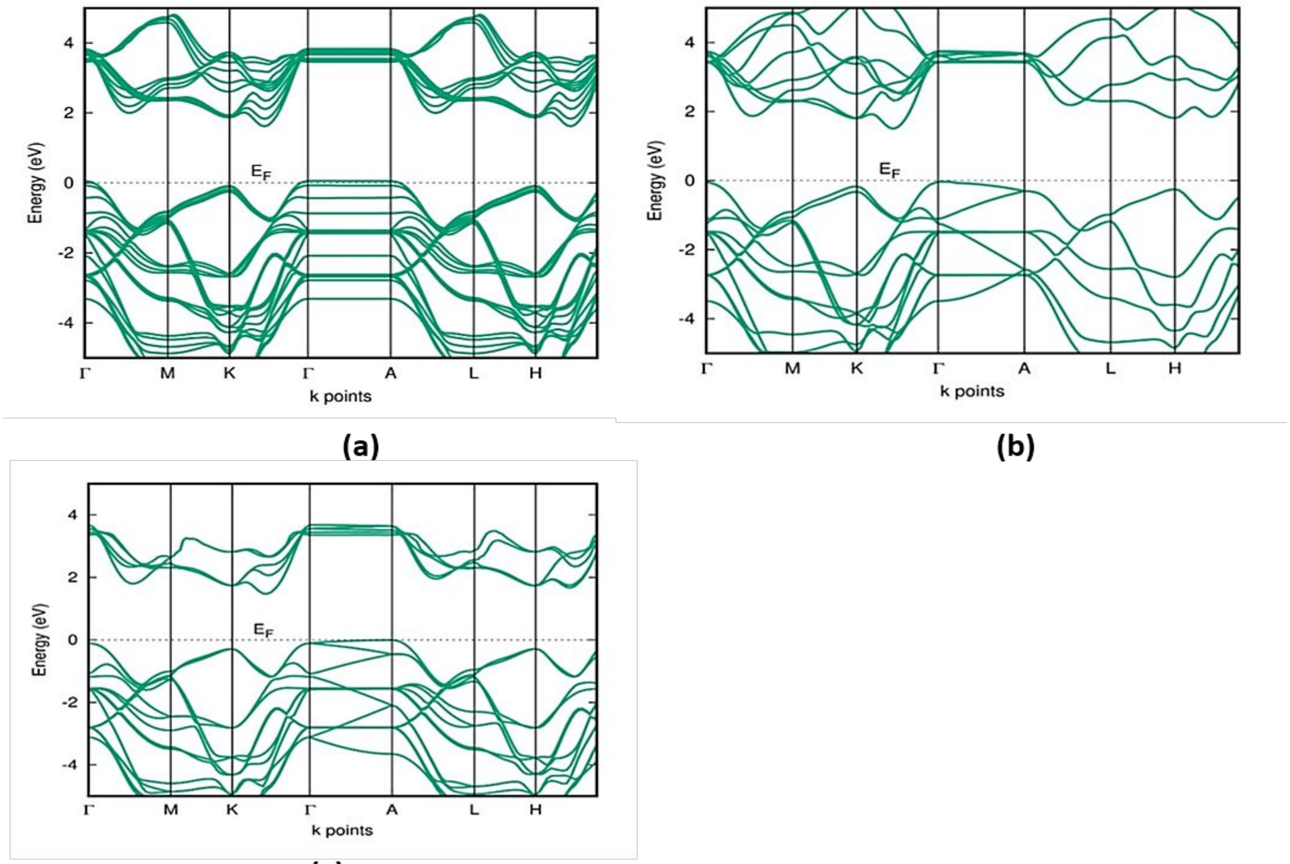

(c)

Figure S1. HSE06 band structure for group A (4T-$MoSe_2$ in (a) 3H_b-$MoSe_2$in (b) and 2R_1-$MoSe_2$ in (c)). We see that the group A polymorphs are semiconductors with a bandgap between 1.6 and 2.0 eV

Table S2 Band gap value and Bandgap types of group A polymorph

Polymorphs	Band gap	Band gap type
**Group-A**		
1H-$MoSe_2$	2 eV	Direct
2H-$MoSe_2$	1.8 eV<br>1.58 eV[36]<br>1.88 eV [37]	Indirect
$3H_a$-$MoSe_2$	2 eV	Direct
$3H_b$ -$MoSe_2$	1.6 eV	Indirect
2T-$MoSe_2$	1.8 eV	Indirect
4T-$MoSe_2$	1.6 eV	Indirect
$2R_1$-$MoSe_2$	1.6 eV	Indirect

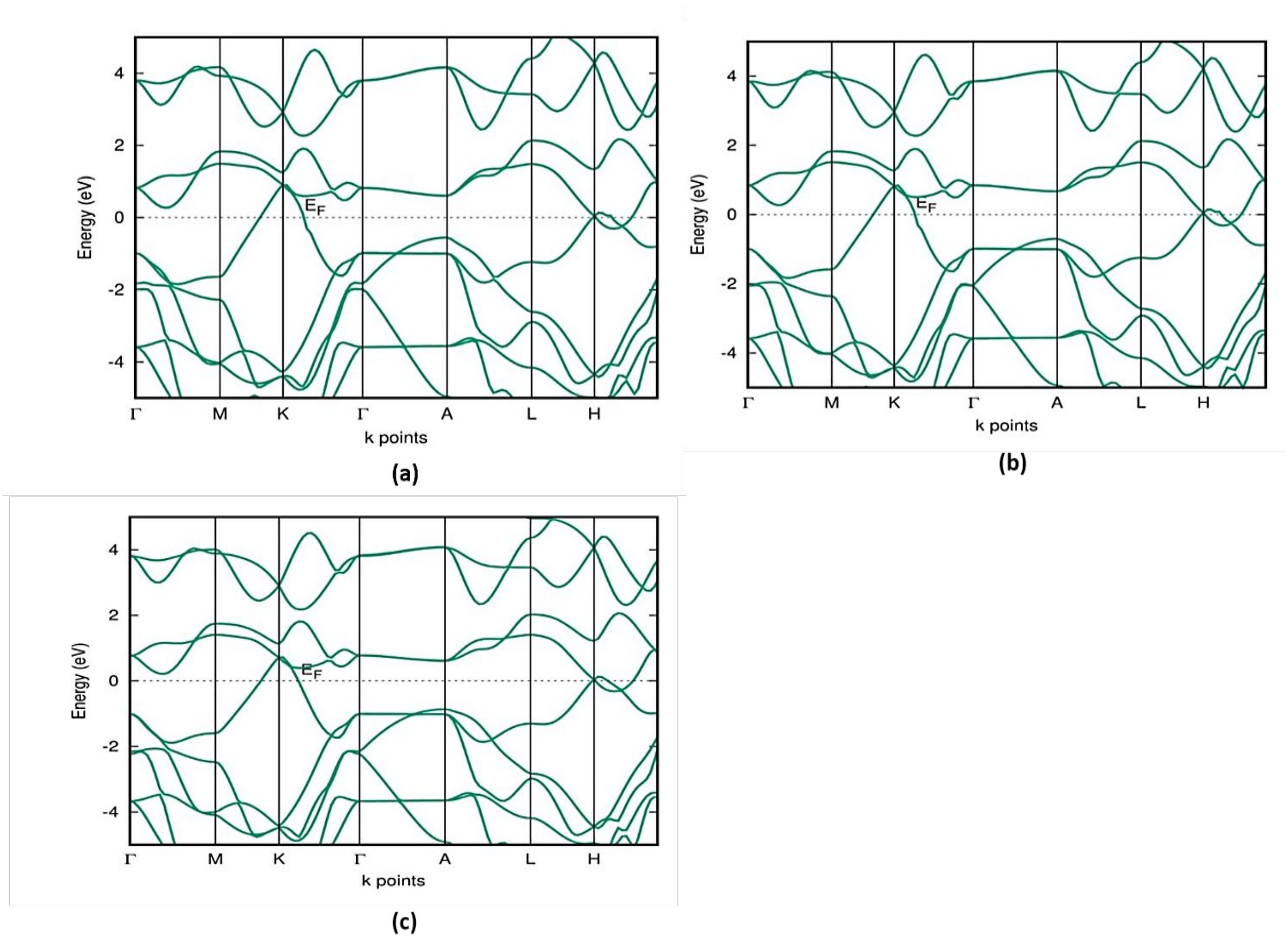

Figure S2. HSE06 band structure for group B, $1T_1$-$MoSe_2$ in (a), $1T_2$-$MoSe_2$ in (b), and $2R_2$-$MoSe_2$ in (c). All the group B polymorphs are metallic.

4. Vibrational study

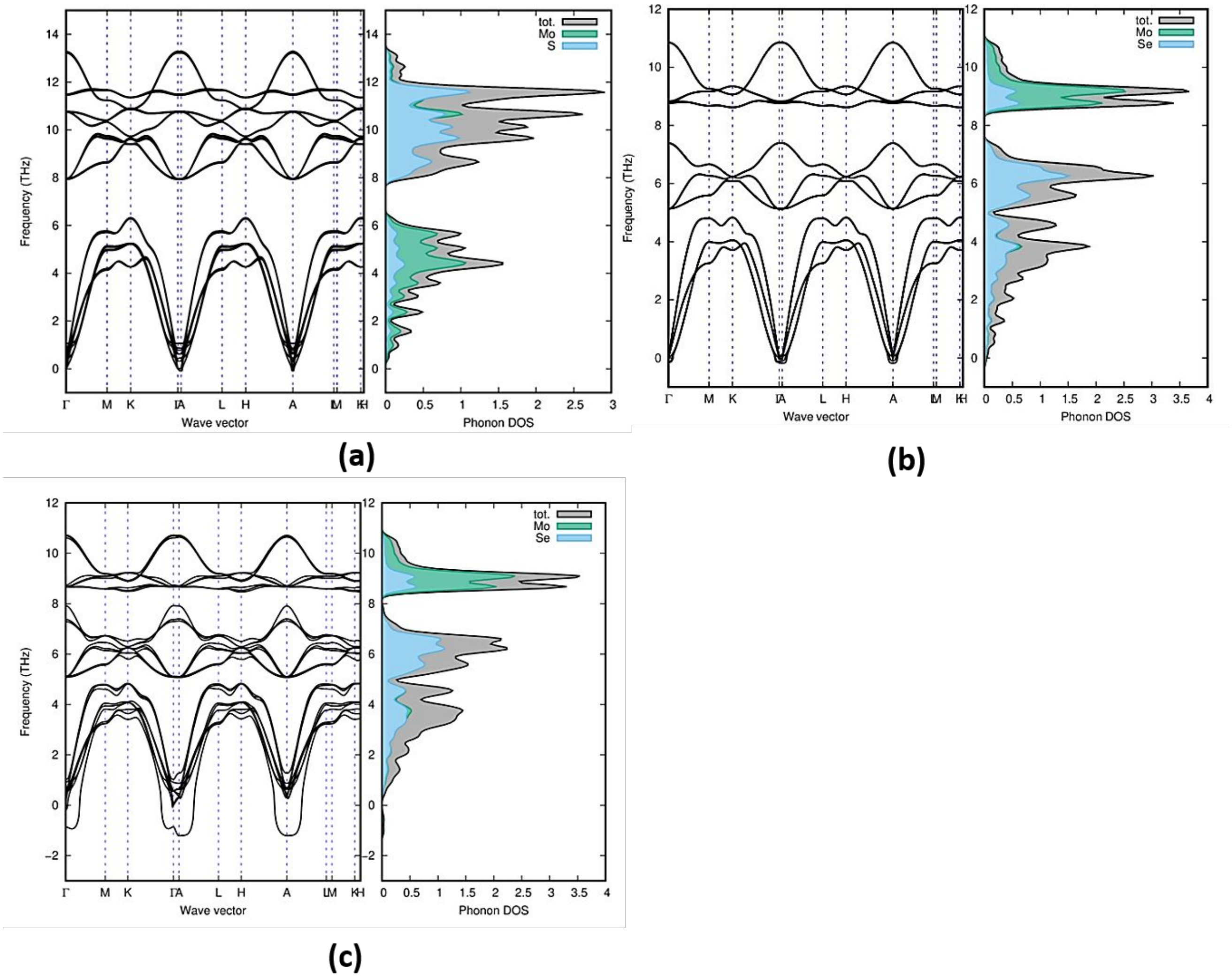

Figure S3. Phonon dispersion and phonon density of states for 4T-$MoSe_2$ (a), $3H_a$-$MoSe_2$ (b), and $2R_1$-$MoSe_2$ (c) polymorphs in group A.

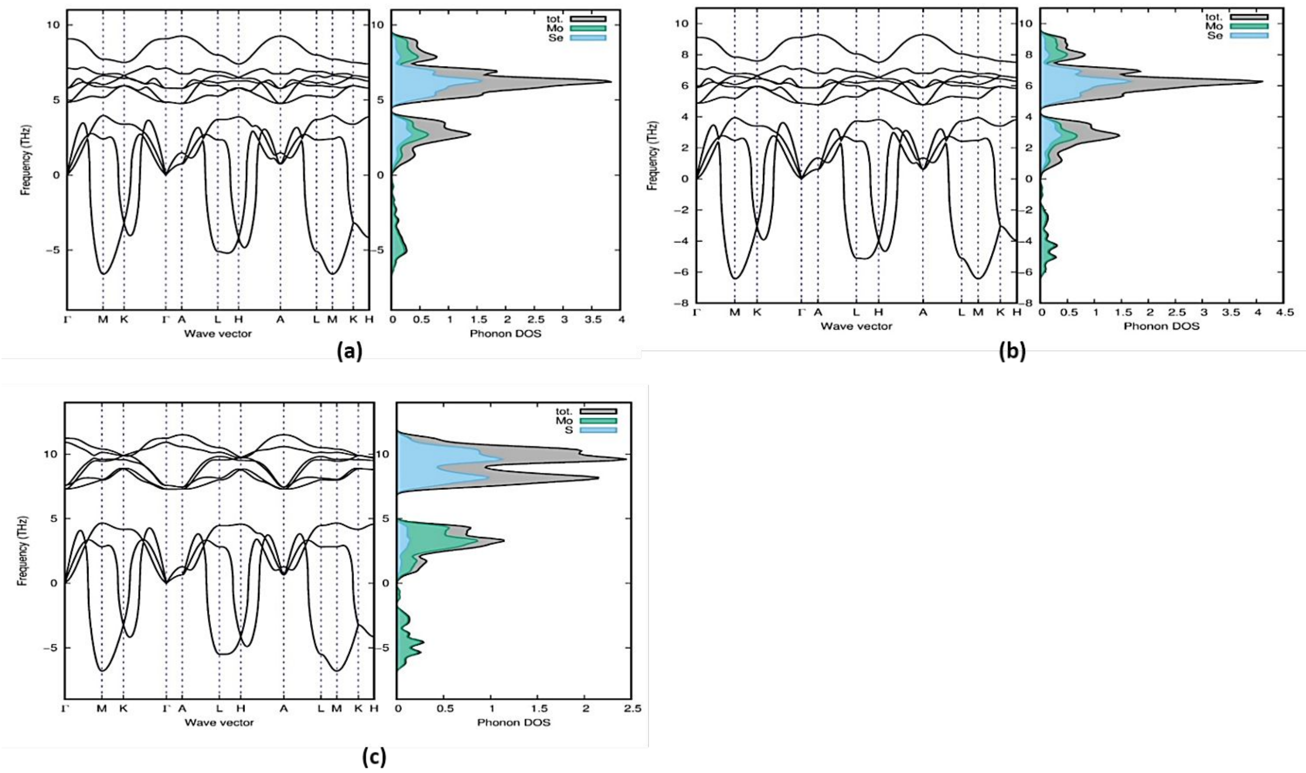

Figure S4. Phonon dispersion and phonon density of states for $1T_1$-$MoSe_2$ (a), $1T_2$-$MoSe_2$ (b), and $2R_2$-$MoSe_2$ (c) polymorphs in group B. All the group B polymorphs are holds negative frequencies, which means dynamically unstable

5. Mechanical stability

Table S3. The calculated single-crystal elastic constants *C*ij (in GPa), bulk modulus B (in GPa), shear modulus G (in GPa), Poisson's ratio ν, Young's modulus E (in GPa). Subscript V indicates the Voigt bound, R indicates the Reuss bound and H indicates the Hill bound

Polymorph	$3H_a$ - $MoSe_2$	4T-$MoSe_2$	$2R_1$ - $MoSe_2$	$1T_1$ $MoSe_2$	$1T_2$ $MoSe_2$	3T - $MoSe_2$	$2R_2$- $MoSe_2$
B_V	34.37	-6028.34	56.95	67.62	14.18	58.37	55.20
B_R	-10.37	3211.09	21.61	49.13	13.78	41.82	29.75
B_H	11.99	-1408.62	39.28	58.37	13.98	50.10	42.48
G_V	17.43	-3492.28	40.63	53.14	66.64	56.80	43.23
G_R	0.09	1.61	18.84	24.30	40.02	39.65	13.93

G_H	8.76	-1745.33	29.73	38.72	53.33	48.22	28.58
ν_V	0.283	0.257	0.212	0.189	-0.415	0.133	0.190
ν_R	0.504	0.500	0.162	0.288	-0.238	0.140	0.297
ν_H	0.206	0.062	0.198	0.228	-0.340	0.136	0.225
E_V	44.74	-8781.18	98.47	126.33	77.91	128.67	102.85
E_R	0.28	4.83	43.79	62.60	61.00	90.39	36.16
E_H	21.14	-3705.56	71.23	95.14	70.44	109.53	70.04

6. METHOD OF CALCULATION

Table S4. Used k-grid size for the HSE calculation and supercell size for the phonon calculation for the involved polymorphs.

**Polymorphs**	**k-grid**	**Supercell size**	**Polymorphs**	**k-grid**	**Supercell size**
**Group-A**			**Group-B**		
1H-$MoSe_2$	8 ×8 × 8	4 × 4 × 1	$1T_1$-$MoSe_2$	12 × 12 ×12	4× 4 × 2
2H-$MoSe_2$	8 × 8 × 8	4 × 4 × 1	$1T_2$-$MoSe_2$	8 × 8 × 8	4 × 4 × 2
$3H_a$-$MoSe_2$	8 × 8 × 8	4 × 4 × 1	3T-$MoSe_2$	12 × 12 ×12	3 × 4 × 1
$3H_b$-$MoSe_2$	8 × 8 × 8	4 × 4 × 1	$2R_2$-$MoSe_2$	8 × 8 × 8	3 × 4 × 1
2T-$MoSe_2$	8 × 8 × 8	4 × 4 × 1			
4T-$MoSe_2$	8 × 8 × 8	3 × 4 × 1			
$2R_1$-$MoSe_2$	8 × 8 × 8	3 ×4 × 1			
